# Altered ability to access a clinically relevant control network in patients remitted from major depressive disorder

**DOI:** 10.1002/hbm.24559

**Published:** 2019-03-12

**Authors:** Caroline A. Figueroa, Joana Cabral, Roel J. T. Mocking, Kristina M. Rapuano, Tim J. van Hartevelt, Gustavo Deco, Paul Expert, Aart H. Schene, Morten L. Kringelbach, Henricus G. Ruhé

**Affiliations:** ^1^ Department of Psychiatry, Academic Medical Center University of Amsterdam Amsterdam The Netherlands; ^2^ Brain Imaging Center Academic Medical Center Amsterdam The Netherlands; ^3^ School of Social Welfare University of California Berkeley Berkeley California; ^4^ Department of Psychiatry University of Oxford Oxford United Kingdom; ^5^ Life and Health Sciences Research Institute (ICVS), School of Medicine University of Minho Braga Portugal; ^6^ Center for Music in the Brain Aarhus University Aarhus Denmark; ^7^ Department of Psychological and Brain Sciences Dartmouth College Hanover New Hampshire; ^8^ Center for Brain and Cognition, Computational Neuroscience Group, Department of Information and Communication Technologies Universitat Pompeu Fabra Barcelona Spain; ^9^ Institució Catalana de la Recerca i Estudis Avançats (ICREA) Barcelona Spain; ^10^ Department of Neuropsychology Max Planck Institute for Human Cognitive and Brain Sciences Leipzig Germany; ^11^ School of Psychological Sciences Monash University Melbourne Australia; ^12^ Centre for Mathematics of Precision Healthcare Imperial College London London United Kingdom; ^13^ Department of Mathematics Imperial College London London United Kingdom; ^14^ Department of Psychiatry Radboud University Medical Center Nijmegen The Netherlands; ^15^ Donders Institute for Brain, Cognition and Behavior Radboud University Nijmegen The Netherlands

**Keywords:** cognitive control, dynamic FC, functional networks, major depressive disorder, resting‐state fMRI

## Abstract

Neurobiological models to explain vulnerability of major depressive disorder (MDD) are scarce and previous functional magnetic resonance imaging studies mostly examined “static” functional connectivity (FC). Knowing that FC constantly evolves over time, it becomes important to assess how FC dynamically differs in remitted‐MDD patients vulnerable for new depressive episodes. Using a recently developed method to examine dynamic FC, we characterized re‐emerging FC states during rest in 51 antidepressant‐free MDD patients at high risk of recurrence (≥2 previous episodes), and 35 healthy controls. We examined differences in occurrence, duration, and switching profiles of FC states after neutral and sad mood induction. Remitted MDD patients showed a decreased probability of an FC state (*p* < 0.005) consisting of an extensive network connecting frontal areas—important for cognitive control—with default mode network, striatum, and salience areas, involved in emotional and self‐referential processing. Even when this FC state was observed in patients, it lasted shorter (*p* < 0.005) and was less likely to switch to a smaller prefrontal–striatum network (*p* < 0.005). Differences between patients and controls decreased after sad mood induction. Further, the duration of this FC state increased in remitted patients after sad mood induction but not in controls (*p* < 0.05). Our findings suggest reduced ability of remitted‐MDD patients, in neutral mood, to access a clinically relevant control network involved in the interplay between externally and internally oriented attention. When recovering from sad mood, remitted recurrent MDD appears to employ a compensatory mechanism to access this FC state. This study provides a novel neurobiological profile of MDD vulnerability.

## 1 INTRODUCTION

Major depressive disorder (MDD) is a severe psychiatric disease, which globally accounts for the greatest loss of years due to disability (Smith, 2014). This high impact of MDD is related to its high incidence and recurrence rates, particularly in patients with multiple previous episodes (Bockting, Spinhoven, Wouters, Koeter, & Schene, 2009). However, this vulnerability during MDD *remission* has scarcely been studied from a level of intrinsic brain connectivity. Elucidating neural vulnerability factors in remitted recurrent MDD (rrMDD) could facilitate the development of effective prediction tools or improve preventive treatments against MDD recurrence (Fischer, Keller, & Etkin, 2016).

Previous studies have linked MDD to disrupted resting‐state functional connectivity (FC) in several resting‐state networks (RSNs) and systems, including frontal networks (FNs), regulating cognitive control and attention, the default mode network (DMN), involved in internal attention, the salience network (SN) and frontostriatal pathways, both involved in salience detection and emotion (Furman, Hamilton, & Gotlib, 2011; Kaiser, Andrews‐Hanna, Wager, & Pizzagalli, 2015; Menon, 2011; Mulders, van Eijndhoven, Schene, Beckmann, & Tendolkar, 2015). During remission between episodes, MDD *vulnerability* might specifically relate to a failure of control systems to downregulate DMN activity, with the SN as switching hub between the networks (Marchetti, Koster, Sonuga‐Barke, & De Raedt, 2012; Servaas et al., 2017). However, the scarce research that has been conducted in *remitted*‐MDD previously examined “static” FC, representing mean connectivity over a period of scanning. Instead, growing evidence shows that brain activity at rest is not stable during the scan, but slowly wanders through a repertoire of time‐varying, but reoccurring, states of coupling among brain regions (Cabral, Kringelbach, & Deco, 2017; Deco, Jirsa, & McIntosh, 2011; Hansen, Battaglia, Spiegler, Deco, & Jirsa, 2015). Dynamic‐FC (dFC) analysis allows characterizing these reoccurring FC states.

dFC changes during tasks (Sakoglu et al., 2010) and resting‐state (Damaraju et al., 2014; Jin et al., 2017; Kaiser et al., 2016) have been associated with psychiatric disorders and with reduced behavioral/cognitive performance in healthy subjects (Cabral, Vidaurre, et al., 2017; Jia, Hu, & Deshpande, 2014; Madhyastha, Askren, Boord, & Grabowski, 2015). For MDD specifically, findings from recent studies suggest aberrations in dFC involving DMN, (Wise et al., 2017) frontal, and SN areas (Demirtaş et al., 2016; Kaiser et al., 2015). However, it remains unclear whether this persists during MDD remission.

The best way to characterize dFC remains under debate (Hutchison et al., 2013). Although the sliding‐window analysis is most commonly used to calculate successive dFC matrices (Sakoglu et al., 2010), the window size affects the temporal resolution, challenging its validity (Deco et al., 2017; Hindriks et al., 2016; Hutchison et al., 2013; Lindquist, Xu, Nebel, & Caffo, 2014; Preti, Bolton, & Ville, 2016; Shine et al., 2015). In the current study, we instead use a recently developed method, the Leading Eigenvector Dynamics Analysis (LEiDA), which calculates dFC at the instantaneous level (for each recorded frame), and allows identifying patterns of blood oxygen level dependent (BOLD) phase coherence, or FC states, that reoccur over time both within and across scanning sessions (Cabral, Vidaurre, et al., 2017). Operating in the temporal domain, this method allows characterizing recurrent FC states in terms of probabilities of occurrence, duration and transition profiles on a subject‐by‐subject level, which allows statistical comparisons between groups. Previously, the dynamical properties of recurrent FC states have been shown to relate with cognitive performance in healthy participants (Cabral, Vidaurre, et al., 2017). Here, we used the same LEiDA method to investigate whether there are specific configurations of dFC that differentiate between antidepressant‐free rrMDD and controls without personal and familial MDD history. We compared differences in occurrence, duration, and switching profiles of FC states both after neutral and sad mood induction. We hypothesized to find alterations in dFC, particularly involving the frontoparietal network, the DMN, and the SN, which would be influenced by sad mood (Cohen, 2017; Harrison et al., 2008).

## 2 METHODS

### 2.1 Participants

After approval by the local Medical Ethical Committee and written informed consent, 62 rrMDD patients with ≥2 depressive episodes as defined by the Structured Clinical Interview for DSM disorders (SCID), in stable remission for ≥2 months according to DSM IV criteria, and 41 healthy controls were scanned. Hamilton Depressive Rating Scale (HDRS‐17) scores, an observer rated MDD‐symptom scale to assess depression severity, were ≤7 (Rush et al., 1986). Patients were antidepressant free for ≥8 weeks. Controls did not have a personal or familial history for psychiatric disease (assessed by SCID). All participants were aged 35–65 years. We excluded participants with alcohol/drug dependency; psychotic or bipolar disorder; predominant anxiety disorder; severe personality disorder; electroconvulsive therapy ≤2 months before scanning; history of severe head trauma; neurological disease; severe general physical illness; and no Dutch/English proficiency. rrMDD patients and controls were matched for age, sex, educational level, and working class. Participants were recruited through identical advertisements in freely available online and house‐to‐house papers, posters in public spaces and from previous studies in our and affiliated research centers (Mocking et al., 2016). See Appendix S1 (Supporting Information) for information about psychiatric comorbidity of rrMDD patients.

### 2.2 Mood‐induction paradigm

As described in more detail in previous work regarding this mood induction (Figueroa et al., 2017; Mocking et al., 2016), before the scan, participants described with as much detail as possible a memory which they regarded as neutral, (e.g., doing the dishes) and one which they regarded as being among the saddest in their life (e.g., losing a job and death of a relative). In addition, participants chose one neutral and one sad fragment of music from 10 different fragments. Memories were scripted in key sentences for display on the screen in the MRI scanner. During memory display, we played the chosen neutral or sad music. Participants were asked to rate their current mood on a scale of 0 to 10 (0 being extremely sad; 10 extremely happy) after the neutral mood induction, after the neutral resting‐state scan and before and after the sad mood induction. After the sad resting‐state scan, the most extreme sadness was rated. The gap between the neutral and sad mood induction, in which participants completed other fMRI tasks was ±125 min, including a 30 min break (Mocking et al., 2016). We designed the sad mood induction to be at the end of all fMRI scanning, as it might have been too stressful for participants to continue fMRI scanning and tasks after the sad mood induction (Figure S1, Supporting Information).

### 2.3 Image acquisition and analyses

A 3 Tesla Philips Achieva XT scanner (Philips Medical Systems, Best, the Netherlands), equipped with a 32‐channel SENSE head coil, was used to obtain the images. A high‐resolution T1‐weighted 3D structural image was acquired using fast‐field echo for anatomical reference (220 slices; repetition time (TR): 8.3 ms; TE: 3.8 ms; field of view (FOV): 240 × 188; 240 × 240 matrix; voxel size: 1 × 1 × 1 mm^3^). Functional images were acquired with T2*‐weighted gradient echo planar imaging (EPI) sequences. Participants were instructed to close their eyes without falling asleep. The scans comprised 210 volumes of 37 axial slices (TR: 2,000 ms; TE: 27.6 ms; FOV: 240 × 240; 80 × 80 matrix; voxel size = 3 × 3 × 3 mm^3^). Slices were oriented parallel to the anterior commissure‐posterior commissure (AC–PC) transverse plane and acquired in ascending order with a gap of 0.3 mm.

### 2.4 Preprocessing

We preprocessed functional MRI data with FMRIB's Software Library (FSL, https://www.fmrib.ox.ac.uk/fsl). We used the default parameters of an imaging preprocessing pipeline on all participants: Multivariate Exploratory Linear Optimized Decomposition into Independent Components (MELODIC 3.14). MELODIC is usually used to conduct an independent component analysis but here we only used it for motion correction and high‐pass filtering of the data. This pipeline further consisted of motion correction using FMRIB's linear image registration tool (MCFLIRT) (Jenkinson, Bannister, Brady, & Smith, 2002); nonbrain removal using brain extraction tool (BET) (Smith, 2002); spatial smoothing using a Gaussian kernel of full width half maximum (FWHM) 5 mm; grand‐mean intensity normalization of the entire 4D dataset by a single multiplicative factor applying a standard high‐pass temporal filtering (Gaussian‐weighted least‐squares straight line fitting, with σ_t_ = 50.0 s and σ_f_ = 0.02 Hz). The EPI images were coregistered to the T1‐ weighted structural images, and the T1‐weighted images were coregistered to standard MNI space.

We used the Anatomical Automatic Labeling (AAL) atlas (Tzourio‐Mazoyer et al., 2002) to parcelate the MNI brain into *N =* 90 cortical and subcortical noncerebellar brain areas and the BOLD signals were then averaged over all voxels belonging to each brain area. The BOLD signals in each of the 90 brain areas were subsequently band‐pass filtered between 0.02 and 0.1 Hz (using a second‐order Butterworth filter), partially discarding the high frequency components associated with cardiac and respiratory signals (>0.1 Hz), and focusing on the most meaningful frequency range of resting‐state fluctuations (Biswal, Yetkin, Haughton, & Hyde, 1995; Cabral, Vidaurre, et al., 2017; Glerean, Salmi, Lahnakoski, Jaaskelainen, & Sams, 2012).

### 2.5 Dynamic functional connectivity

**Figure 1:**
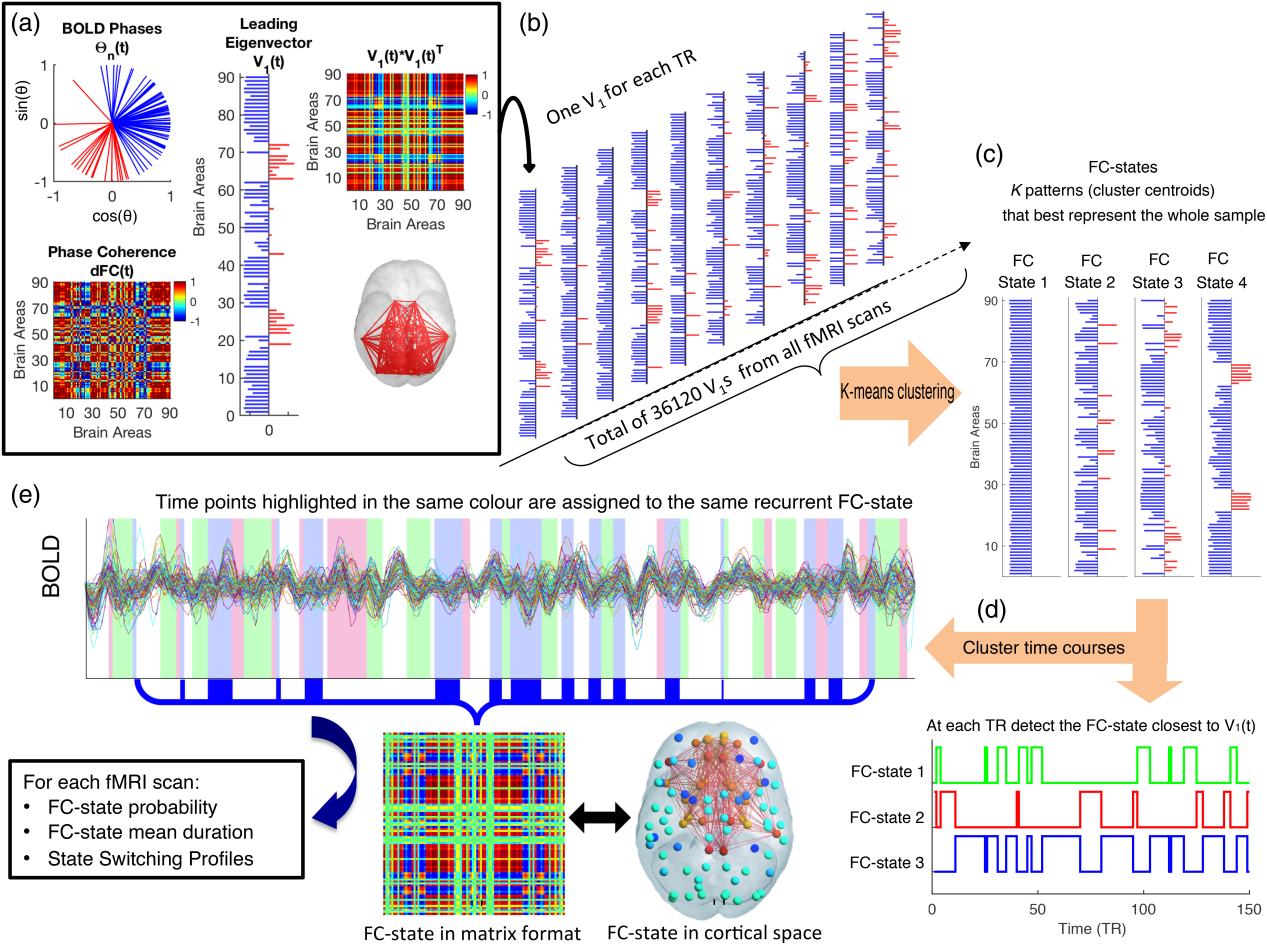
Time courses of recurrent functional connectivity (FC) states obtained with Leading Eigenvector Dynamics Analysis (LEiDA). (a) Leading eigenvector of BOLD phase coherence (top left) phase portrait of BOLD signal phases at a given time point *t* in all *N* = 90 brain areas (bottom left). The *N* × *N* phase coherence matrix, dynamic‐FC (dFC(*t*)) indicates for each pair of areas how coherent they are, where 1 (red) means full synchrony and −1 (blue) indicates a phase difference of 180° (middle). The leading eigenvector *V*_1_(*t*) of this matrix is a *N* × 1 vector that, when multiplied by its transpose *V*_1_(*t*) × *V*_1_(*t*) reveals the dominant pattern of the dFC(*t*) matrix (top right). Note that the product of elements with the same sign (be they negative or positive) is always positive, so negative values in the matrix are between pairs with different signs. The signs of the elements in *V*_1_ (red/blue) are used to divide brain areas into communities according to their BOLD phase, which can be visualized in cortical space (here links between the areas with positive elements in *V*_1_ are plotted in red). (b) The leading eigenvectors *V*_1_ are obtained for each time point from all fMRI scans in all subjects, resulting in a large sample of 36,120 leading eigenvectors. (c) This sample is partitioned into a reduced number of *K* clusters (here we varied *K* between 2 and 20). Each cluster is represented by a central vector, which may not necessarily be a member of the data set. We take these cluster centroid vectors as representing recurrent patterns of BOLD phase coherence, or FC states. (d) To obtain the FC‐state time courses, we select, at each TR, the cluster number to which *V*_1_(*t*) is the most similar. The cluster time courses (illustrated as color‐shaded bars, over a single fMRI session) are then used to calculate, for each scan, the probability, mean duration, and the state‐switching probabilities of each FC state (bottom). For illustration, the FC state assigned to the blue‐shaded time points are displayed as an *N* × *N* matrix (outer product) and as a network in cortical space (here lighter and darker colors show stronger and weaker coherence, respectively, within the positive [yellow‐red] versus the negative [cyan‐blue] communities) [Color figure can be viewed at http://wileyonlinelibrary.com]

We used BOLD phase coherence connectivity (Deco et al., 2017; Deco & Kringelbach, 2016; Glerean et al., 2012; Ponce‐Alvarez et al., 2015) to obtain a time‐resolved dFC tensor, with size *N* × *N* × *T,* where *N* = 90 is the number of brain areas considered in the current parcellation scheme (see Section 2.4), and *T =* 210 is the number of recording frames in each scan. We first estimated the phase of the BOLD signals in all *N* = 90 areas over time*,* θ(*n*,*t*)*,* using the Hilbert transform, which expresses a signal *X* as *X*(*t*) *= A*(*t*)cos(θ(*t*))*,* where *A*(*t*) is the instantaneous amplitude, and θ(*t*) is the instantaneous phase. In Figure 1a**,** we represent the portrait of all *N* = 90 BOLD phases at time *t*, which can be represented in Cartesian coordinates with cos(θ(*n,t*)) in the horizontal axis and sin(θ(*n,t*)) in the vertical axis. Each entry dFC(*n,p,t*) contains to the BOLD phase coherence between brain areas *n* and *p* at time *t,* obtained using the following equation:

$$\mathrm{dFC}\left(n,p,t\right)=\cos\left(\theta \left(n,t\right)-\theta \left(p,t\right)\right)$$

where cos() is the cosine function. dFC(*n,p,t*) is 1 if two areas *n* and *p* have synchronized BOLD signals at time *t*, and dFC(*n,p,t*) is 0 if the BOLD signals are orthogonal (with a phase difference of 90°).

### 2.6 FC states

To identify recurrent patterns in the dFC, LEiDA considers, at each time *t*, only the leading eigenvector *V*_1_(*t*) of each dFC(*t*) (i.e., the one associated with the largest magnitude eigenvalue), which captures only the dominant pattern of FC, instead of considering the whole matrices (Cabral, Vidaurre, et al., 2017). As can be seen in Figure 1a, this vector contains *N* elements (each representing one brain area) and their sign (positive or negative) can be used to separate brain areas into two communities according to their BOLD‐phase relationship (Newman, 2006). When all elements of the largest magnitude eigenvector, *V*_1_(*t*), have the same sign, it means all BOLD phases are following in the same direction with respect to the orientation determined by *V*_*1*_(*t*), which is indicative of a global mode governing all BOLD signals. If instead the first eigenvector *V*_1_(*t*) has elements of different signs (i.e., positive and negative), the BOLD signals follow different directions with respect to the leading eigenvector, which we use to divide brain areas into two “communities” according to their BOLD phase relationship (see Figure 1a). Moreover, the magnitude of each element in *V*_1_(*t*) indicates the “strength” with which brain areas belong to the communities in which they are placed (Newman, 2006). The dominant FC state can also be represented back into matrix format (*N* × *N*) by computing the outer product *V*_1_ *V*_1_^*T*^, which is visually similar to the original dFC(*t*) matrix, despite being a matrix of Rank 1 (i.e., a matrix that is obtained from a single vector). Since *V* and –*V* represent the same vector orientation, we use a convention ensuring that most of the elements have negative values.

Conveniently, eigenvectors can be represented in cortical space by representing each element as a sphere placed at the center of gravity of the corresponding brain area, and scaling the color of each sphere according to the value of the corresponding eigenvector element. As such, areas with coherent BOLD signals are colored alike (yellow‐to‐red for the smallest community and cyan‐to‐blue for the largest community), where lighter colors (cyan/yellow) indicate stronger contributions and darker colors (blue/red) weaker contributions. To highlight the network formed by the smallest community of brain areas, we plot links between the corresponding areas. For example, Figure 1 (bottom right) shows an FC state represented in cortical space, where the BOLD signals can be divided into two modes: a larger set of brain areas (cyan areas) and a smaller functional network (orange/red areas) formed by areas whose BOLD signal is coherent but phase shifted with respect to the other community. In the matrix format, all links between pairs of areas with the same sign (be it positive or negative) have a positive value (red). Negative values in the matrix format (blue) correspond to links between areas with different signs in the eigenvector.

In this work, we aimed to explore whether there are specific FC configurations that differentiate rrMDD patients from controls. To do so, we first cluster all the samples of FC states into a reduced number of recurrent patterns, applying a *k*‐means clustering algorithm to all leading eigenvectors *V*_1_(*t*) across all subjects (rrMDD and controls in neutral and sad mood, resulting in 210 × 86 × 2 = 36,120 leading eigenvectors) (see Figure 1b). The clustering divides the samples into *k* clusters (each representing a recurrent FC configuration), with higher *k* revealing more rare and more fine‐grained network configurations. Although there is not a consensus regarding the number of FC states revealed by fMRI (and whether FC states can be discretized in the first place), the number of RSNs reported in the literature generally falls between 7 and 17, depending on the selected criteria (Damoiseaux et al., 2006; Yeo et al., 2011).

Here, we do not aim to determine the optimal number of FC states, but rather to explore if there is an FC state that significantly differs (consistently) between remitted patients and controls, even if that FC state is different from previously reported networks and/or occurs only rarely in time. As such, we varied *k* (number of clusters) over a wide range between 2 and 20 and for each *k*, examined how each FC state changed between groups. Importantly, the clustering assigns a single FC state to each fMRI time frame, as highlighted by the shaded bars in Figure 1d. The clustering obtained for each value of *k* is obtained independently of each other and thus represent independent models of FC configuration space.

### 2.7 Between‐group comparisons

**Figure 2:**
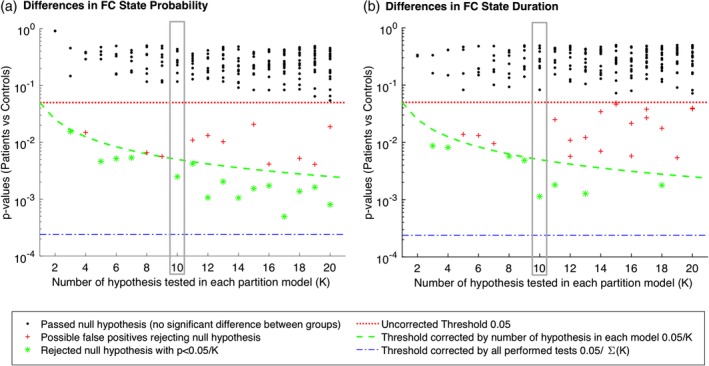
Significance of between‐group differences in functional connectivity (FC)‐state probability and lifetime as a function of *k*. For each partition of the sample into *k* = 2 to 20 FC states, we plot the *p*‐values associated with the between‐group comparison between patients and controls in both FC‐state probability (a) and lifetimes (b). We find that, although most FC states do not show significant differences between groups (black dots falling above the 0.05 threshold, in red), for all *k* > 2, there is one FC state that consistently falls below (or very near) the corrected threshold by the number of clusters (<0.05/*k*, green dashed line). The *p*‐values marked as red crosses pass the standard threshold (<0.05) but do not survive the correction for multiple comparisons within each partition model (>0.05/*k*) and are hence considered false positives. The blue dashed line refers to the threshold correction if all hypothesis were independent across models, losing statistical power (i.e., increased probability of false negatives) when the hypothesis are not independent, as it is the case when considering the whole sample of tests performed [Color figure can be viewed at http://wileyonlinelibrary.com]

**Figure 3:**
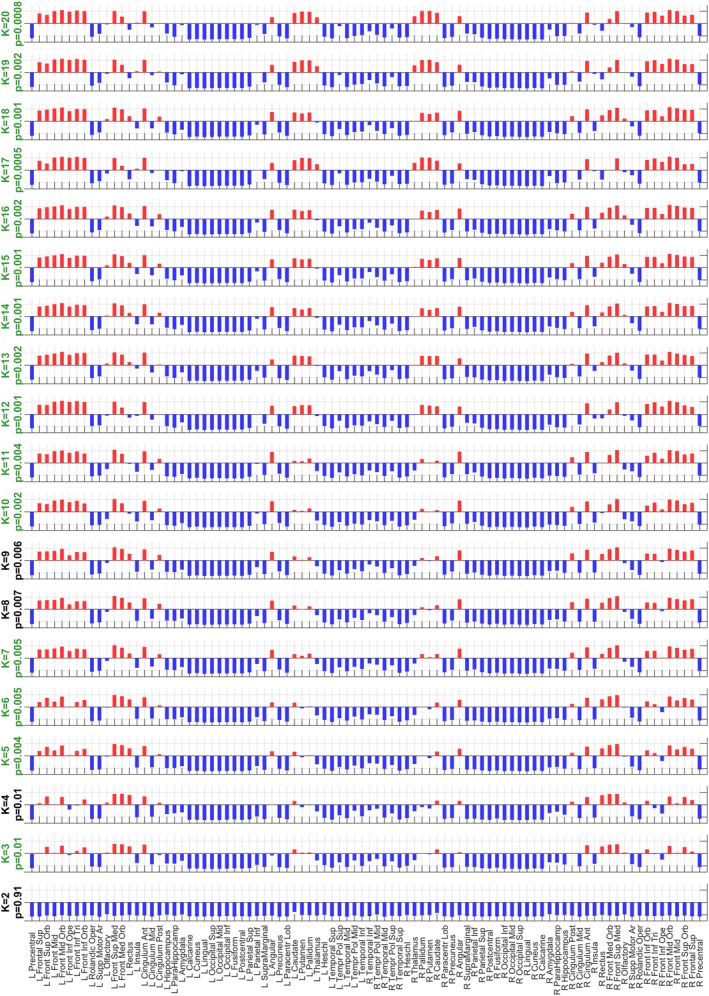
Consistency of the functional connectivity (FC) state with the most significant differences in probability of occurrence between remitted patients and controls over partition models. After clustering all eigenvectors into *k* = 2 to 20 clusters, we consistently find, for all *k* > 2, that the FC state most different between groups is a functional network consisting of areas of the frontal cortex coupled (in terms of BOLD phase alignment) with basal ganglia (i.e., caudate, putamen, and pallidum) and the angular gyrus (default mode network [DMN]). Note that the involvement of basal ganglia in the relevant network becomes more pronounced for more fine‐grained partitions (higher *k*). For all partition models with *k* > 2, the probability of occurrence of these FC states between groups passed the standard significance threshold of 0.05, but only 15 (highlighted with a green title) passed the significance threshold when corrected by the number of states, or independent hypothesis, compared within each partition model [Color figure can be viewed at http://wileyonlinelibrary.com]

To assess how the repertoire of *k* FC states explored during rest varied between groups, we first calculated, for each subject and condition, the probability of occurrence of each FC state (fraction of epochs it occurred throughout the scan duration), the mean duration of each FC state (mean number of consecutive epochs in the same state), the switching frequency (number of transitions per second [Hz]) and the switching profiles (probabilities of switching from a given FC state to another). All values were compared between rrMDD patients and controls, after neutral and sad mood induction using (nonparametric) permutation‐based *t* tests (10,000 permutations). For each FC repertoire obtained by *k*‐means clustering, *k* hypotheses are tested. To correct for multiple comparison, we adjusted the significance threshold to 0.05/*k*, using a Bonferroni correction (green dashed line in Figure 2). We then evaluated the consistency of the FC states that were found to be significantly different between groups across the range of *k* explored (Figures 2 and 3, see Section 3.2). We also report within‐group differences for neutral versus sad mood (rrMDD patients and controls separately) in the Results, Supporting Information. For statistical testing involving clinical and demographic characteristics, we used SPSS version 25. For the LEiDA analysis, we used MATLAB version R‐2017b.

### 2.8 Code availability statement

The LEiDA codes are publicly available at http://github.com/juanitacabral/LEiDA in the folder: “Remission from Major Depression.”

## 3 RESULTS

### 3.1 Sample characteristics

Here, 72 rrMDD patients and 46 controls were initially eligible, of which 62 and 41 were scanned, respectively. Of these participants, we excluded nine rrMDD and six controls because of abnormal brain anatomy and two rrMDD due to technical difficulties (corrupted scans). Then, 51 rrMDD patients and 35 controls were included in the final analysis (Figure S2, Supporting Information). No significant differences were observed between rrMDD patients and controls for sex, age, education, IQ, living situation, employment status, and handedness. rrMDD patients showed higher levels of residual depressive symptoms (HDRS) *p* < 0.001 (Table 1). Comparisons between rrMDD and controls did not change when restricted to the sample selected for the present fMRI analyses.

**Table 1:** Sample characteristics

				Between‐group statistics			
		rrMDD	HC	χ^2^	*t*	*U*	*p*
		(*n* = 62)	(*n* = 41)				
Female	*N* (%)	43 (69.3%)	28 (68.3%)	0.01			0.91
Age	Years; mean (*SD*)	53.7 (7.9)	51.8 (8.1)		1.17		0.25
Education	Levels^a^	0/0/0/4/21/23/14	0/0/0/1/16/17/7	1.49			0.69
IQ	Mean (*SD*)	108 (8.5)	106 (9.9)			878.5	0.14
Living situation	Levels^b^	26/0/18/14/2/0/2	10/0/16/11/4/0/0	6.23			0.18
Employment status	Levels^c^	24/23/15/0	21/16/4/0	3.7			0.16
Handedness	Levels^d^	4/50/4	2/33/4	0.44			0.8
Age of onset	Years; mean (*SD*)	27.2 (11.2)	–				–
Episodes	Median (IQR)	4.0 (2/4/7)	–				–
HDRS	Median (IQR)	2.0 (1/2/5)	1.0 (0/1/1)			644	<0.001

HC = healthy control; HDRS = Hamilton Depression Rating Scale; IQR: interquartile range; LEIDS‐R = Leiden Index Depression Sensitivity‐Revised; rrMDD = remitted recurrent major depressive disorder; RRS = Ruminative Response Scale.

a Level of educational attainment (Verhage, 1964): levels range from 1 to 7 (1 = primary school not finished; 7 = preuniversity/university degree).

b Living situation: alone/living with parents/cohabiting/cohabiting with children/single living with children/other/unknown.

c Employment status: low/middle/high/never worked.

d Handedness = left/right/ambidexter; IQR = interquartile range; χ^2^ = chi‐square test statistic; *p* = *p*‐value; *U* = Mann–Whitney *U* nonparametric test statistic; *t* = independent‐samples *t*‐test statistic. This table was published before (Figueroa et al., 2017).

Both groups reported comparable neutral to positive mood after neutral mood induction (*p* = 0.095). Mood scores (±*SD*) decreased only slightly during neutral resting state (rrMDD: −0.41 ± 0.93, controls: −0.022 ± 0.65), without a group × mood interaction (*p* = 0.36). The sad mood induction significantly decreased mood scores in both groups (rrMDD: −2.13 ± 1.50, controls: −2.15 ± 1.17; *p* < 0.001), also without a group × mood‐induction interaction (*p* = 0.95). The lowest mood reported during the second resting‐state was significantly lower in rrMDD (rrMDD: 4.99 ± 1.88 vs. controls: 5.94 ± 1.39; *p* = 0.027). See Table S1 (Supporting Information) for all mood ratings.

### 3.2 Detection of the most different FC states

The repertoire of FC states obtained depends on the number of clusters determined in the *k*‐means clustering algorithm, with generally a higher number of clusters resulting in more fine‐grained, less frequent and often less symmetric networks. Importantly, here we do not aim to determine the optimal number of FC states explored during rest but instead to search for FC configurations that most significantly and consistently differentiate patients remitted from recurrent MDD from controls. In Figure 2, we show, for each clustering model of the FC state samples into *k* FC state categories, the *k p*‐values obtained from between‐group comparisons in terms of probability and lifetimes. In each model, *k* hypotheses are tested. Thus, to account for the increased probability of false positives, we Bonferroni corrected the significance threshold to 0.05/*k* (green dashed line in Figure 2). We find that, irrespective of the number of clusters selected (as long as *k* > 2), the clustering consistently returns an FC state that significantly differs in probability between patients in remission and controls, falling below (or very near) the threshold corrected by the number of clusters within each partition model (green dashed line in Figure 2 **).**

In detail, of the 19 partition models considered (i.e., with *k* ranging from 2 to 20), 15 solutions revealed an FC state that occurred significantly less in rrMDD compared to controls after correcting for the number of clusters in each repertoire of FC states (*p* < 0.05/*k*). The most significantly different FC states of each partition model are reported in Figure 3 in vector format, where areas with the same color are assigned to the same mode of phase coherence. The 15 FC states that passed the corrected significance threshold, were highly correlated (Pearson's *r* > 0.84 for all pairs of FC states), which indicate that they refer to variant forms of the same underlying FC state, with differences arising from the number of output states constrained by *k*. In terms of duration, this FC state also lasted significantly shorter in seven partition models (*p* < 0.05/k), of which four had overlap with a significant lower probability (see Figure 2). Of note, for *k* = 18, the FC state that lasted significantly shorter in rrMDD patients includes only the subcortical areas (caudate, putamen, pallidus, thalamus, and amygdala) (2.74 ± 0.26 vs. 4.55 ± 0.63 s, *p* = 0.0018, uncorrected). This reveals that more detailed partitions (higher *k*) can further subdivide the FC states, revealing finer grained structures that differentiate between groups (see Figure S3, Supporting Information for dominant FC states from *k* = 2 to 20 and Figure S4, Supporting Information for the variance in each *k*‐means derived FC state for each partition model).

The consistency of our findings for a range of partition models reinforces the existence of a specific pattern of BOLD phase coherence that differentiates patients in remission from controls. For the subsequent analysis, we selected the partition into *k* = 10 FC states, since it returned a repertoire of 10 FC states where only one FC state significantly differed both in terms of probability and lifetime (see Figure 2, blue dots in the gray rectangle in both [a] and [b]), whereas all other nine FC states in this partition model did not show significant differences (*p* > 0.05 for all black dots within the gray rectangles). Moreover, the partition into 10 states is aligned with previous studies in the resting‐state literature (Damoiseaux et al., 2006).

### 3.3 Relevant FC state

**Figure 4:**
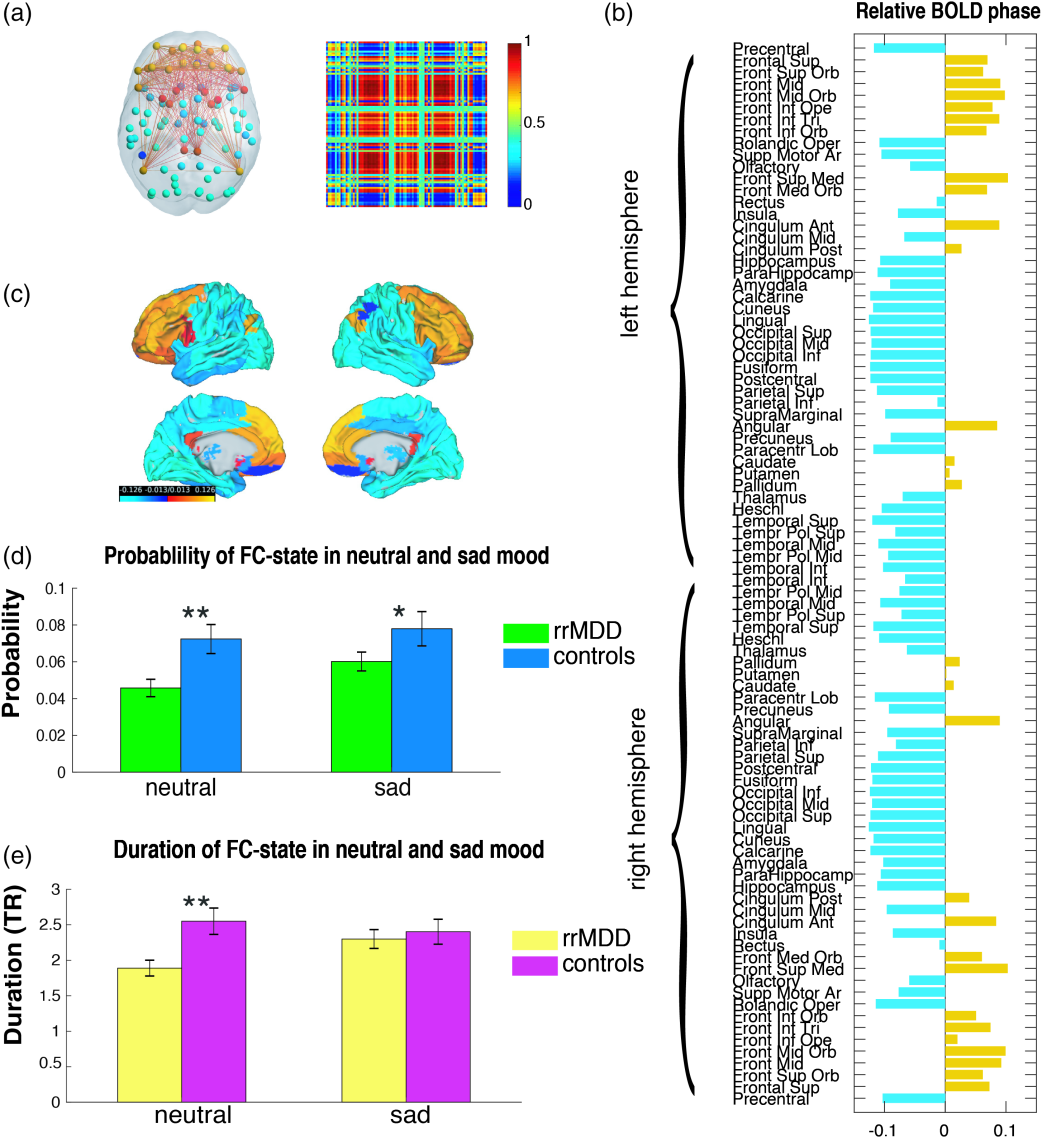
Functional connectivity (FC) state with a significant difference between rrMDD patients and controls (*k* = 10) in probability of occurrence and lifetime during neutral mood. (a) The dominant connectivity state is represented in the cortical space, where functionally connected brain areas (represented as spheres) are colored alike. The spheres colored in yellow, orange, and red, represent areas in the FN, default mode network (DMN), striatum, and SN, which are all positively correlated between each other, but negatively correlated with the rest of the brain (cyan/blue colored spheres). The dominant state is also represented as the eigenvector's outer product, which is a 90 × 90 matrix representing the number of brain areas and red or blue indicate positive or negative BOLD phase synchronization between them. (b) Contribution of different brain areas to the dominant FC state. Bars in yellow represent areas in the FN, DMN, striatum, and SN and bars in light blue represent the rest of the brain. The magnitude of values indicates the “strength” with which brain areas belong to the FC state. (c) The significant FC state rendered on the cortex. (d) Differences in probability of occurrence of this state between rrMDD patients and controls (4.58 ± 0.47% vs. 7.24 ± 0.79%, respectively, *p* = 0.0022 in neutral mood and 6.0 ± 0.51% vs. 7.80 ± 0.93%, *p* = 0.049 in sad mood). (e) Differences in lifetime of this state between rrMDD patients and controls (3.78 ± 0.11 vs. 5.10 ± 0.18 s, *p* = 0.0020 and 4.60 ± 0.26 vs. 4.80 ± 0.36, *p* = 0.32 in sad mood). ** Significant group difference after correcting for multiple comparisons. * Significant group difference before correcting for multiple comparisons. Abbreviations: MDD = major depressive disorder; rrMDD = remitted recurrent MDD; TR = repetition time [Color figure can be viewed at http://wileyonlinelibrary.com]

Our analysis revealed an FC state, which consistently appeared less and lasted shorter in rrMDD compared to controls in neutral mood (Figure 4 **;** FCstate.mp4**;** Figure S3, Supporting Information). This underrepresented FC state consists of an extensive network including frontal (dorsolateral prefrontal cortex [DLPFC] and fronto‐orbital cortex), DMN (posterior cingulate cortex, angular gyrus, and medial prefrontal cortex [MPFC]), subcortical (the dorsal striatum [Str] [caudate, putamen] and pallidus), and SN areas (anterior cingulate cortex, frontal operculum). We further indicate this FC state as the “FN‐DMN‐Str‐SN state.” See Figure S5, Supporting Information for the overlap of this FN‐DMN‐Str‐SN state with RSNs defined by previous whole brain parcellations (Choi, Yeo, & Buckner, 2012; Yeo et al., 2011).

For the selected clustering model (*k* = 10), the FN‐DMN‐Str‐SN state occurred less frequently (4.58 ± 0.47% compared to 7.24 ± 0.79% of the time, *p* = 0.0025, uncorrected*,* Hedge's *g* = 0.67; Hedge's *g* = 0.67; medium to large effect size) and lasted shorter when dominant (3.78 ± 0.11 vs. 5.10 ± 0.18 s, *p* = 0.0011, Hedge's *g* = 0.71; medium to large effect size) (Figure 4; FCstate.mp4).

**Figure 5:**
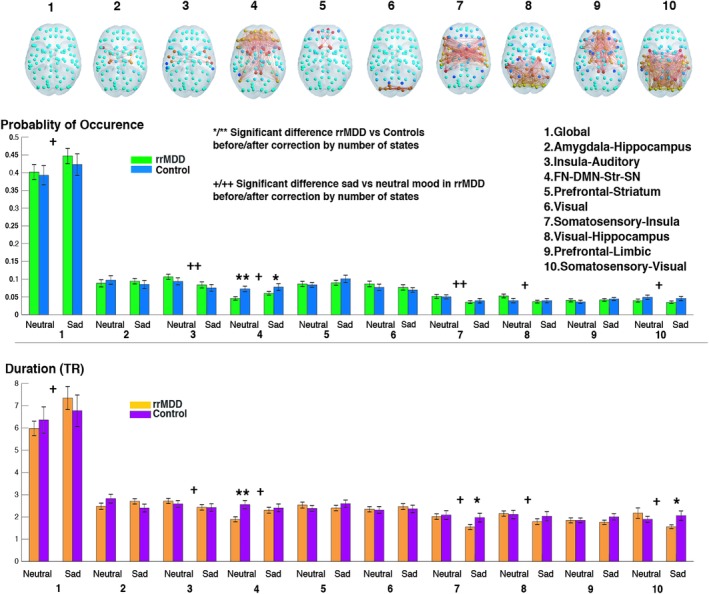
Differences in probability and duration of each functional connectivity (FC) state between and within groups for neutral and sad mood conditions for *k =* 10. FC states are represented in the cortical space, where functionally connected brain areas (represented as spheres) are colored alike. The spheres colored in yellow/red represent areas that are all positively correlated between them, but negatively correlated with the rest of the brain (cyan/blue colored spheres). Names of FC states were defined by loading network maps into neurosynth, http://neurosynth.org/decode/, a platform for large‐scale, automated synthesis of functional magnetic resonance imaging (fMRI) data. In the case of the state that was significantly different between groups, (FC state 4), we additionally compared the FC state with resting‐state networks (RSNs) defined by a whole‐brain parcellation scheme (Choi et al., 2012; Yeo et al., 2011) (Figure S5, Supporting Information). Of note, when considering other clustering solutions other FC states may be identified. */** Significant difference between rrMDD and controls before/after correcting for number of states (*p < 0.05, **p < 0.05/k), +/++ Significant within‐group difference for rrMDD, neutral versus sad before/after correcting for number of states (+*p* < 0.05, ++*p* < 0.05/*k*). Abbreviations: DMN = default mode network; FN = frontal network; MDD = major depressive disorder; rrMDD = remitted recurrent MDD; SN = salience network; Str = striatum; TR = repetition time [Color figure can be viewed at http://wileyonlinelibrary.com]

In Figure 5 **,** we show the full repertoire of FC states that are returned by LEiDA when choosing *k* = 10. This reveals different network configurations that appear, dissolve, and reoccur in all subjects during the scan, with the state of global coherence showing not only the highest probability but also the highest variability. Notably, these networks overlap with previously described RSNs (Choi et al., 2012; Yeo et al., 2011) and with cognitive states based on a large‐scale automatic synthesis of human functional neuroimaging data (Yarkoni, Poldrack, Nichols, Van Essen, & Wager, 2011).

### 3.4 Correction for residual symptoms

For the FN‐DMN‐Str‐SN state, we examined whether the differences between rrMDD and controls in duration and lifetime remained significant after correcting for residual depressive symptoms (HDRS scores). In a regression analysis with group (rrMDD or control) and HDRS as independent variables and probability/lifetime as the dependent variable, the group differences remained significant (*p* = 0.007 and *p* = 0.006, respectively). This indicates that current level of depressive symptoms likely does not explain the group differences.

### 3.5 Effect of sad mood induction on FC states

During sad mood, of the 19 partition models considered, the FN‐DMN‐Str‐SN state had a lower probability of occurrence in three clustering solutions of a higher order (*k* = 17, 19, and 20), that is, when more subdivisions in FC states are made (Figure S3, Supporting Information), after correction for multiple comparisons (*p* < 0.05/*k*). Furthermore, significantly shorter lifetimes were found for this FC state in partition models of a lower order (*k* = 5 and 6) (Figure S3, Supporting Information).

As can be seen in Figure 4d,e, for *k* = 10, the probability of being in the FN‐DMN‐Str‐SN state in sad mood was only significantly different between groups before correcting by *k* (6.0 ± 0.51% compared to 7.80 ± 0.93% of the time, respectively, *p* = 0.048, uncorrected, Hedge's *g* = 0.41, indicating a small to medium effect size). Further, no differences were detected in terms of duration of FC states (4.60 ± 0.26 vs. 4.80 ± 0.36 s, respectively, *p* = 0.32, uncorrected, Hedge's *g* = 0.10, indicating a small effect size).

### 3.6 Group × mood interactions

We examined if there were significant group × mood interaction effects for the probability of occurrence and mean lifetime of the FN‐DMN‐Str‐SN state for *k* = 10 by means of a repeated measures ANOVA. We found no significant interaction effects for this FC pattern for probability, *F* = 0.634 *p* = 0.425, but there was a significant interaction effect for lifetime, *F* = 4.32, *p* = 0.041. These interaction effects did not significantly change when correcting for residual symptoms in a repeated measures ANCOVA, *p* = 0.259 and *p* = 0.024, respectively.

### 3.7 Within‐group differences in FC states for neutral versus sad mood

Remitted‐MDD showed lower probabilities and lifetimes for sad versus neutral mood in multiple FC states (Results/Figure S6, Supporting Information). For controls, there were no within‐group differences for neutral versus sad mood.

### 3.8 Switching frequencies

Overall, mean switching frequencies (number of switches/second) did not differ between rrMDD and controls for all clustering solutions (from *k =* 2 to 20) in neutral and in sad mood (all *p*‐values >0.05).

### 3.9 Switching probabilities

**Figure 6:**
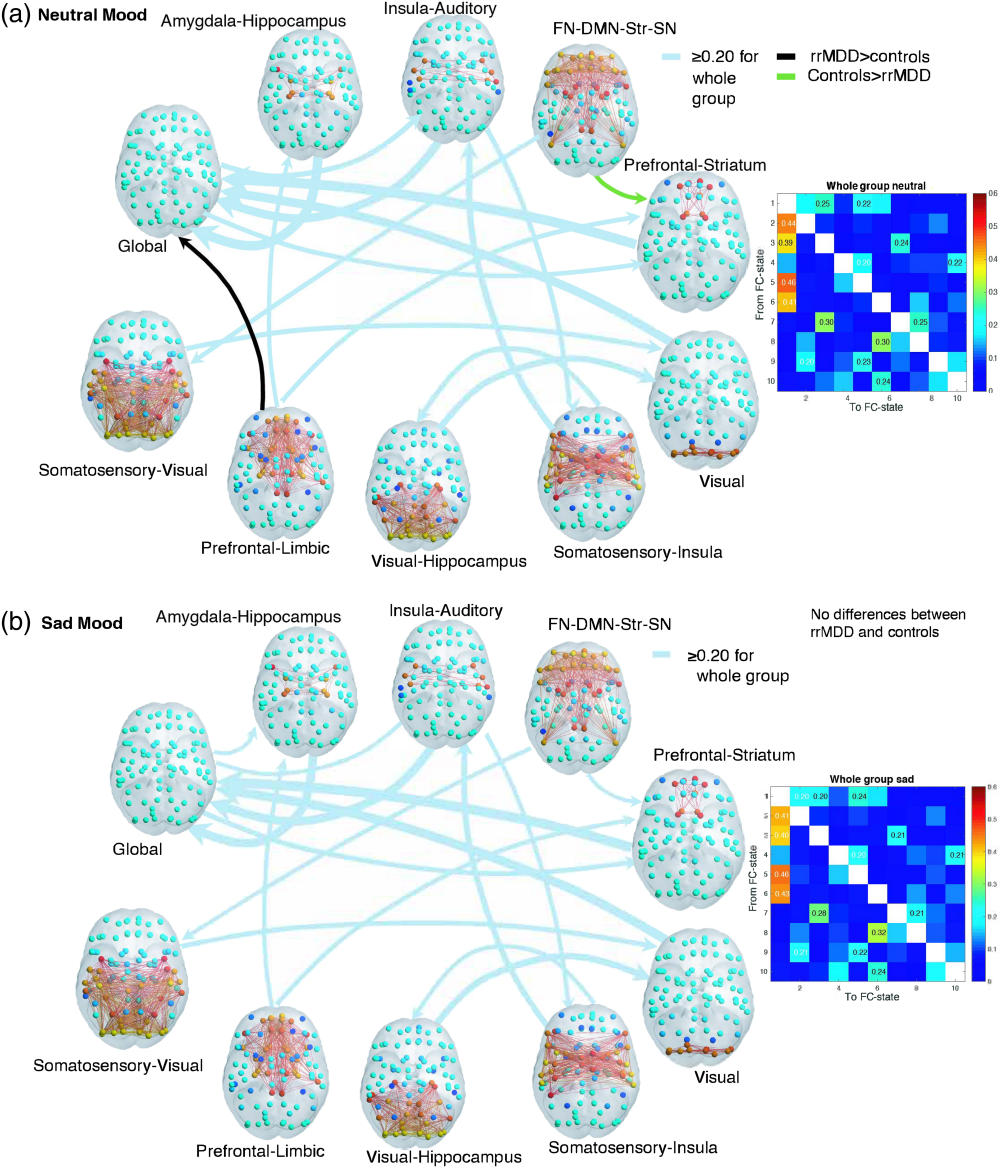
(a,b) Switching probabilities for the whole group and differences between rrMDD and controls, for (a) neutral and (b) sad mood. Switching probabilities for the whole group are shown above a threshold of 20% probability of switching to show more frequent switches. The whole group switching matrices (titled “whole group neutral” and “whole group sad”) indicate the probability of, being in a given functional connectivity (FC) state (rows), transitioning to any of the other states (columns) for the whole group. FC states are represented in the cortical space, where functionally connected brain areas (represented as spheres) are colored alike. The spheres colored in yellow/red represent areas that are all positively correlated between them, but negatively correlated with the rest of the brain (cyan/blue colored spheres). The light blue arrows from and to the FC states indicate the whole group switching probabilities, scaled to the magnitude of probability of switching. Significantly different transitions after correcting for *k* = 10 (*p* < 0.05/10) for rrMDD compared to controls are illustrated in this figure, with the black arrow representing the transition that occurs with higher probability in rrMDD and in green the one that occurs with higher probability in controls (note these arrows have not been scaled according to magnitude of probability of switching). Transitioning differences between groups were calculated using a permutation‐based two sample *t* test with 10,000 permutations. rrMDD patients showed a lower probability of switching from the FN‐DMN‐Str‐SN state to the prefrontal–striatum state (16.5 vs. 23%, *p* = 0.004), and a higher probability to switch from the prefrontal–limbic state to the global state (6 vs. 1%, *p* = 0.003). We identified no significant between group differences in sad mood after correction for *k* = 10 (*p* > 0.05/k). Abbreviations: DMN = default mode network; FN = frontal network; MDD = major depressive disorder; rrMDD = remitted recurrent MDD; SN = salience network; Str = striatum [Color figure can be viewed at http://wileyonlinelibrary.com]

We examined the transition patterns between FC states in detail for the selected partition model (*k* = 10) by calculating the probability of, being in a given FC state, switching to any of the other FC states. In Figure 6, we illustrate the general switching pattern for the whole group, with blue arrows indicating the switches that exceed a probability of 20% of occurring (numeric values reported in the switching matrix on the right). On a whole‐group level, the most common switches were observed from FC states 2, 3, 5, and 6 toward the global FC state (first column in the switching matrix), suggesting that after being coherent in BOLD phase for some time, the areas involved in these functional networks realign their BOLD phases with the global signal, returning to a state of global BOLD phase coherence, which is the most prevalent FC state.

Comparing the switching patterns between rrMDD and controls, we found that, even when rrMDD patients displayed the FN‐DMN‐Str‐SN state, they showed a significantly lower probability of switching from this state to the prefrontal–striatum state compared to controls (16.5 vs. 23%, *p* = 0.004, uncorrected, *p* = 0.04 corrected by *k*; Figure 6a, green arrow). In addition, we also detected a higher probability to switch from the prefrontal–limbic state to the state of global BOLD coherence (6% rrMDD vs. 1% controls, *p* = 0.002, uncorrected, *p* = 0.02 corrected by *k*; Figure 6a, black arrow). Conducting a deeper analysis of the transition profiles shown in Figure 6, we can see that the trajectory from the FN‐DMN‐Str‐SN state before returning to the state of global BOLD coherence occurs preferentially via the prefrontal–striatum state in controls. In rrMDD, this trajectory appears disrupted, suggesting that rrMDD patients not only have decreased ability to access the FN‐DMN‐Str‐SN network but also to switch from this to the prefrontal–striatum state.

### 3.10 Effect of sad mood on switching profiles

The overall switching pattern after sad mood induction for the whole group was similar to the switching pattern during neutral mood (Figure 6b). During sad mood, for *k* = 10, we no longer identified significant differences in switching probabilities between groups (all *p* > 0.05/*k*, *k* = 10). See Figure S7, Supporting Information for differences with *p* < 0.05.

### 3.11 Within‐group differences for neutral versus sad mood

Differences in switching probabilities were observed both for rrMDD and controls for neutral versus sad mood, which we describe in the Results and Figures S7 and 8a,b (Supporting Information).

## 4 DISCUSSION

This study investigated differences in FC states reoccurring over time during resting‐state in rrMDD patients not taking antidepressants compared with never depressed controls. We identified decreased ability in rrMDD patients to access an FN‐DMN‐Str‐SN state, consisting of frontal, DMN, striatum, and SN areas during neutral mood. Our study provides a new framework for detecting network abnormalities associated with vulnerability during MDD remission.

The FN‐DMN‐Str‐SN state, which consistently differed between rrMDD and controls, with a medium to high effect size, reveals an extensive network of clinically relevant areas including key areas from the DMN (posterior cingulate cortex (PCC) and MPFC), executive network (dorsolateral prefrontal cortex (DLFPC)), and SN (anterior cingulate cortex (ACC)) (Figure S4, Supporting Information). These networks have been identified as affected in MDD before, and together form the “triple network,” a model employed for understanding affective and neurocognitive dysfunctions across multiple disorders (Menon, 2011). The extensive FN included in this FC state consists of areas activated during cognitive control and flexible switching of attention from internal thought processes to the external environment, with the DLPFC being particularly relevant (Japee, Holiday, Satyshur, Mukai, & Ungerleider, 2015).

Additionally, the FC state includes a large DMN component. Dominance of the DMN over networks involved in cognitive control has been associated with depressive symptoms (Knyazev, Savostyanov, Bocharov, Tamozhnikov, & Saprigyn, 2016), rumination (Hamilton et al., 2011), and might be related to depressive recurrence, though this has not been empirically tested (Marchetti et al., 2012). Furthermore, the state included areas of the SN, a network that has been proposed to play a key role in switching brain activity between introspective, ruminative DMN functions and task‐based executive networks functions (Menon & Uddin, 2010). Finally, the state included the dorsal striatum (caudate and putamen) and globus pallidus, structures involved in the focusing (and filtering) of cortical input (Grillner, Hellgren, Menard, Saitoh, & Wikstrom, 2005). Abnormal functioning of frontostriatal pathways might additionally lead to maladaptive regulation of emotions (Drevets, 2007), contributing to anhedonia and rumination in MDD (Furman et al., 2011).

Taken together, our results suggest that, patients remitted from recurrent MDD, show decreased ability to access an FC state in neutral mood regulating cognitive control (FN/SN) to diminish negative self‐referential processes (DMN) and effectively regulate emotions (SN/corticostriatal pathways) (Marchetti et al., 2012).

Additionally, we observed a lower probability in rrMDD to switch from the FN‐DMN‐Str‐SN state to a smaller prefrontal–striatum state. Interestingly, on a group level, the prefrontal–striatum state has a high probability of switching to the state of global BOLD coherence. It has been suggested that the more frequently occurring global brain states allow for a greater range of either integration or segregation between neural networks and brain areas, that is, more flexible switching to different brain states (Nomi et al., 2017). This greater neural flexibility might facilitate cognitive flexibility (Kringelbach, McIntosh, Ritter, Jirsa, & Deco, 2015; Nomi et al., 2017). As an additional measure of neural flexibility, in a post hoc supplementary analysis, we calculated the entropy (Shannon, 1948) for *k* = 10 and the FN‐DMN‐Str‐SN state separately (Discussion, Supporting Information). We found that the entropy associated with the FN‐DMN‐Str‐SN state was significantly decreased in patients in neutral mood**.** This indicates a higher predictability of occurrence (Wang, 2008) of this FC state in rrMDD patients, supporting the idea that this brain state occurs in a less flexible manner.

After the sad mood induction, the duration of the FN‐DMN‐Str‐SN state increased in rrMDD but stayed similar in controls (significant group × mood interaction). This suggests that rrMDD have a decreased ability to access this state in neutral mood but manage to recruit this state during sad mood (albeit slightly less than controls). This differential increase supports our hypothesis that this FC state is relevant for processes associated with affect and emotion in rrMDD.

We speculate that this increase might reflect a compensatory mechanism in rrMDD to regulate brain activity. This could be an attempt (which might or might not be successful) to regulate negative self‐referential and emotional processing when sad mood is induced. In controls, this FC state is already more easily activated in neutral mood and therefore regulation of brain activity during sad mood and in daily life might be more automatic and less effortful for never depressed subjects. This is corroborated by the fact that this FC state occurs more flexibly (as measured by the entropy) in controls during neutral mood than in remitted‐MDD (but not during sad mood, see Results, Supporting Information). Therefore, we hypothesize that an inability to access this state during neutral mood might reflect increased difficulty for rrMDD patients to effectively and flexibly handle daily life stressors and changes in affect (Van der Lande et al., 2016). This hypothesis needs to be tested by future research. Of note, we additionally observed within‐group differences only in rrMDD for sad versus neutral mood (Results, Supporting Information), indicating that the induction of sad mood (i.e., recall of the saddest life experience) affects brain network dynamics more strongly in patients in remission compared to controls.

Of note, though rrMDD reported higher severity of residual depressive symptoms, this did not explain group differences in probability or lifetime of the FN‐DMN‐Str‐SN state. Additionally, rrMDD reported higher levels of sadness than controls before and after the sad mood induction (although both groups' mood decreased equally during the sad mood induction). Overall, it thus seems that these symptoms did not contribute to group differences in the ability to access the FN‐DMN‐Str‐SN state in neutral or sad mood. This could indicate that these dynamic FC differences represent more of a trait effect of depression than a state effect associated with depressive symptomatology.

Interestingly, previous studies in MDD have also found increased dynamic FC in areas overlapping with the FN‐DMN‐Str‐SN state: between the frontoparietal network and DMN (Kaiser et al., 2016) within the DMN (Wise et al., 2017) and between DMN (MPFC) and the insula (Kaiser et al., 2016); related to levels of self‐report rumination (Kaiser et al., 2016). This suggests that dynamic changes in FC in these areas might be particularly important for depression and a ruminative thinking style. However, these studies included acute MDD patients and did not use a mood‐induction procedure, which makes it difficult to compare results. In remitted‐MDD, Foland‐Ross, Cooney, Joormann, Henry, & Gotlib, 2014 found that *during* both a sad mood induction and automatic mood regulation by positive autobiographical recall, remitted participants exhibited a *decrease* in activation in the left ventrolateral prefrontal cortex and cuneus, which are both involved in autobiographical memory processing (Foland‐Ross et al., 2014). However, since this study examined brain activation this is also challenging to compare. Further, we examined resting‐state dynamic FC during the recovery of sad mood, and not during the actual mood induction. It should be examined further how induction of sad mood or stress changes dynamic FC.

Results of this study are novel because our approach differs from common static‐FC analyses, which previously identified aberrations in MDD in networks assumed to be temporally stable over the whole recording time, whereas we here show differences in networks that reoccur and dissolve over time. Of note, the FN‐DMN‐Str‐SN state is only dominant during a small proportion of time. However, it consists of clinically relevant areas implicated in cognitive control and emotional/self‐referential processing and occurred less, especially during neutral mood, in rrMDD patients at high risk for recurrence. Importantly, we found that this FC state consistently differed between groups after multiple comparisons correction, largely independent of which clustering solution was chosen (Figure S3, Supporting Information). Further, using this approach, we found that an FC state of areas traditionally belonging to spatially defined RSNs derived from static‐FC analysis forms a separate network of increased coherence over time. This emphasizes transient dysfunctional interactions *between* multiple networks involved in psychological functioning, as opposed to unistructural or single network abnormalities in the pathophysiology of MDD.

Although this is still unclear, the more a functional network is accessed, the more stable it might become during rest because it reinforces underlying structural connections, perhaps trough Hebbian learning mechanisms (Martin & Morris, 2002). For instance, it has been shown that cognitive training over time does not only alter FC but occurs alongside changes in the structural connectome (Caeyenberghs, Metzler‐Baddeley, Foley, & Jones, 2016). Here, healthy controls might have accessed the FN‐DMN‐Str‐SN state more throughout life, which might be associated with a lower risk of occurrence of a depressive episode. If this hypothesis is true, it could be examined whether interventions such as cognitive control training (Hoorelbeke & Koster, 2017) or neurofeedback (Enriquez‐Geppert, Huster, & Herrmann, 2017), which allows participants to regulate brain processes/states in real time (Zilverstand, Sorger, Zimmermann, Kaas, & Goebel, 2014), could increase the occurrence and duration of this FC state.

Interestingly, two previous studies on dFC in *acute* MDD also found alterations in similar networks involving DMN/frontoparietal and SN areas, albeit using different methods (Demirtaş et al., 2016; Kaiser et al., 2016). Kaiser et al. (2016) used a sliding‐window analysis, which has limitations related to window size, and Demirtaş et al. (2016) used instantaneous FC, more comparable to our study. Our approach of focusing on the dominant FC state has the advantage of being more robust to high‐frequency noise, as recurrences of the same pattern are more clearly detected (Cabral et al., 2017; Cabral, Vidaurre, et al., 2017). Merit for future studies lies in examining whether FC states are altered when MDD patients change from a remitted to a depressed state, and whether FC alterations predict short‐ and long‐term MDD recurrence.

The state that occurred most in both groups was a state of global coherence of BOLD phases. This global state shows the greatest variability of all FC states and might therefore allow for a greater range of correlations between areas to form, thereby functioning as a baseline state from which other FC states are organized (Nomi et al., 2017). However, this FC state might relate to what is commonly described as the “global signal,” defined as the time series of signal intensity averaged across all brain voxels, composed of both neural and nonneural signals (Murphy & Fox, 2017). In this study, movement was corrected using MCFLIRT but no further artifact rejection was carried out. In particular, we chose not to regress out motion parameters or use global signal regression, a procedure which has been suggested to reduce motion‐based signals (Power et al., 2014), though this remains a topic of debate. Furthermore, we did not correct for direct physiological measures nor did we use COMPCOR (Behzadi, Restom, Liau, & Liu, 2007). Not removing these factors could affect our results. It has recently been argued that including/removing this global signal might produce different complementary insights into the brain's functional organization (Murphy & Fox, 2017). The functional properties of this global state of coherence merit further examination.

### 4.1 Strengths and limitations

The main strength of our study is that we have a large sample of antidepressant‐free patients in remission from at least two episodes of MDD, which allows investigating biomarkers for patients at risk of MDD excluding possible medication effects. Further, using a method to characterize FC at the instantaneous level, we identify FC states that, despite occurring with relatively low probability and duration, govern the pattern of BOLD phase coherence recurrently and consistently across subjects, which could be otherwise missed with analysis over longer time windows. Importantly, instead of focusing on single pairwise connections between regions, the identification of recurrent brain‐wide FC patterns allows characterizing FC states on a subject‐by‐subject level, whose properties can subsequently be statistically compared between groups. Although we only examined partition models from *k* = 2 to 20 clusters, we show that results can be robust across a range of partition models, with differences arising from the granularity inherent to the number of selected clusters. The FC states identified in the current work are strongly constrained by the selected parcellation atlas (AAL). Due to the anatomical basis of this atlas and since all areas have different and relatively large sizes, it might have relatively low BOLD signal homogeneity within its regions compared to other fMRI‐based parcellations, due to mixing of functional signals (Craddock et al., 2013). Therefore, extending this methodological approach to parcellations based on functional homogeneity and with similar size (Craddock et al., 2013; Glasser et al., 2016; Shen, Tokoglu, Papademetris, & Constable, 2013), and/or focusing on more fine‐grained FC states is likely to reveal subdivisions within the FN‐DMN‐Str‐SN state. This would allow to potentially identify specific subsystems driving the activation of the FN‐DMN‐Str‐SN state.

Further, we selected a solution of *k* = 10 for further analysis in detail, because this solution yielded the strongest group difference in dynamic properties. This approach could be critiqued as circular and could thus be considered a limitation of our analyses. However, relying on standard algorithms for evaluating cluster performance (which consistently have a penalty for higher dimensionalities) may not necessarily be the appropriate approach for detecting altered patterns in neuroimaging data. Importantly, independent validation experiments should be conducted to confirm that the core of our findings is reproducible and potentially extendable to patients with MDD (see COBIDAS for guidelines on reproducible research; Nichols et al., 2017).

The unclear functional meaning of FC states remains a limitation in studies of dynamic FC. Moreover, the dynamic nature of FC is likely to occur at a much faster timescale than captured by the BOLD signal (here with a TR of 2 s), with magnetoencephalography (MEG) studies pointing to timescales in the order of 200 ms (Baker et al., 2014; Vidaurre et al., 2016). As such, measures like “FC state duration” must be interpreted with caution, having in mind that the BOLD signals were smoothed below 0.1 Hz. Therefore, long durations of a specific FC state (~10 s) most likely reflect periods of high probability occurring at fast frequencies, rather than expressing a prolonged sustained period of a fixed FC configuration. Analyzing the results from a statistical perspective and comparing between groups, as we do here, allows overcoming this limitation in order to capture the most meaningful between‐group differences in connectivity patterns with inherently faster dynamics. Nevertheless, being able to detect meaningful dFC characteristics differentiating people at risk for MDD from controls reinforces the potential of dFC measures to provide biomarkers for psychiatry.

Further, even though we selected remitted participants that were free of current diagnoses of alcohol/drug dependence, psychotic or bipolar, predominant anxiety or severe personality disorder (all assessed by the SCID), a proportion of participants (41%) had other past or current psychiatric comorbidities. Although we performed a sensitivity analysis correcting for the presence of comorbidity in our group comparison for probability and lifetime for the FN‐DMN‐str‐SN state (see Results, Supporting Information), we cannot rule out that these comorbidities did not partly influence the group differences. An additional limitation is that the sad mood induction was at the end of our scanning paradigm. We took this approach because it might have been too emotionally straining for participants to continue with tasks afterward. However, we cannot rule out order effects and/or additional effects of fatigue after other scanning components.

## 5 CONCLUSION

Using the novel LEiDA approach to examine instantaneous dynamic FC, this study provides new insights on aberrations in dynamic brain network connectivity in remitted MDD patients. This new framework for exploring dynamic FC could potentially be extended to other diseases that have been related to pathological resting‐state connectivity. Overall, our findings suggest reduced ability and flexibility of patients remitted from MDD, but at high risk for recurrence, to access a clinically relevant control network involved in the interplay between emotional and attentional processing and self‐referential attention.

## CONFLICT OF INTEREST

The authors declare no conflict of interest.

## Supporting information


**Appendix S1 Supplementary information**
