# Supplementary material for: Altered ability to access a clinically relevant control network in patients remitted from major depressive disorder
Source: Hum Brain Mapp. 2019 Mar 12;40(9):2771–86. doi: 10.1002/hbm.24559 (PMC6865599; doi:10.1002/hbm.24559)
Supplement: Supplementary file 1 — Appendix S1 Supplementary information [file HBM-40-2771-s001.docx]

Supplementary information

Figueroa CA, Cabral J, Mocking RJT, et al. Altered ability to access a clinically relevant control network in patients remitted from Major Depressive Disorder

Supplementary Figure 1. Mood-induction paradigm 2

Supplementary Figure 2. Disposition of participants 3

Past and current psychiatric comorbidity of rrMDD patients 4

Reasons for exclusion from fMRI analysis 4

Supplementary Table 1. Mood-ratings 4

Supplementary Figure 3. Dominant FC-states emerging in brain activity over time 6

Supplementary Figure 4. variance in each k-means derived FC-state for each partition model. 7

Supplementary Figure 5. Overlap of resting-state networks with the FN-DMN-Str-SN state 7

Supplementary Results 8

FC-state analysis for remitted-MDD and controls separately 8

Supplementary Figure 6. FC-states showing differences in probability/duration in rrMDD 9

Supplementary Figure 7. Switching probablity differences; matrices 11

Supplementary Figure 8. A/B Switching probability differences; figures 12

Supplementary Discussion 13

Entropy for k=10 and for the FN-DMN-Str-SN state separately 13

References 16

##
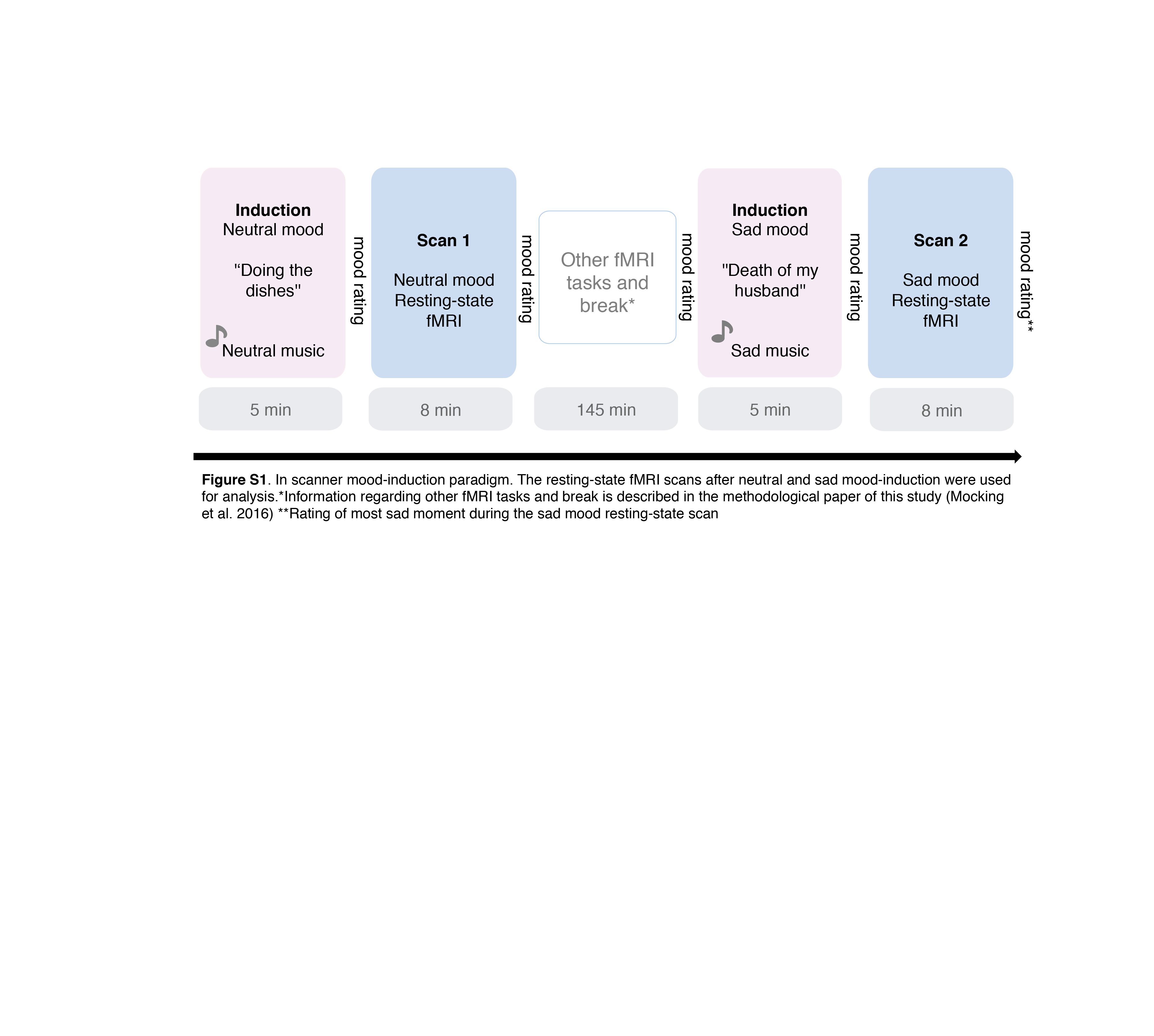
Supplementary Figure 1. Mood-induction paradigm

##

## Supplementary Figure 2. Disposition of participants

**Intake**

N= 125 patients

**Intake**

N= 65 controls

**Screening**

N> 500

Excluded:

- Refusal n=10
- HDRS>7 n=13
- <2 experienced MDD episodes n=4
- No primary depression n=9
- Bipolar disorder n=5
- Current depression n=5
- Current use AD n=2
- Severe head trauma n=1
- Drug dependency n=1
- Unknown n=2

Not fulfilling inclusion criteria (e.g. age, current depressive episode, medication use)

Excluded:

- Refusal n=8
- History of depression n=5
- First degree familial psychiatric disorder n=5
- Unknown n=1

**Inclusion**

N= 46 controls

**Inclusion**

N= 73 patients

**fMRI**

N= 41 controls

**fMRI**

N= 62 patients

Excluded:

- Refusal n=3
- Technical reason n=1
- Possibility of new psychiatric illness n=1

Excluded:

- Refusal n=8
- MRI exclusion criteria n=2
- Start new MDD-episode n=1

**Analysis**

N= 35 controls

**Analysis**

N= 51 patients

Excluded:

- Brain anatomy interfering with connectomics n=6

Excluded:

- Technical difficulties n=2
- Brain anatomy interfering with connectomics n=9

Abbreviations: AD: antidepressants; fMRI: functional Magnetic Resonance imaging; HDRS: Hamilton Depressive Rating Scale; MDD: Major Depressive Disorder

## Past and current psychiatric comorbidity of rrMDD patients

In total, 22 rrMDD patients (41%) had one or more other current or past psychiatric diagnosis (DSM). Six patients had one previous diagnosis: alcohol dependence (n=2) and panic disorder (n=4). Two patients had two past diagnoses: alcohol dependence/religious delusion due to medication, and alcohol dependence/gambling addiction. Nine patients had one current comorbid diagnosis: personality disorder (n=2), social phobia (n=1), anxiety disorder (1), post-traumatic stress disorder (n=3), eating disorder (n=1) and specific phobia (n=1). Four patients had two current comorbid diagnoses: dysthymic disorder/social phobia, panic disorder/eating disorder, panic disorder/anxiety disorder, panic disorder/PTSS. One patient had one current diagnosis; eating disorder, and a past diagnosis: alcohol dependency.

In a sensitivity analysis, using a regression model with group (rrMDD or control) and the presence of comorbidity (yes/no) as independent variables, and probability and lifetime of the FN-DMN-str-SN state as the dependent variable, the group differences remained significant (*p*=0.007 and *p*=0.005, respectively).

## Reasons for exclusion from fMRI analysis

We excluded 9 rrMDD and 6 controls because of abnormal brain anatomy and 2 rrMDD due to technical difficulties (corrupted scans). Seven rrMDD patients and 4 controls were excluded from the fMRI analyses because the coregistration with the AAL could not be performed accurately due to variations in brain anatomy of these patients. Specifically, there was a mismatch with the size of the ventricles, resulting in >50% of the caudate region appearing in the CSF. Further, two rrMDD and 2 controls were excluded because of abnormal brain anatomy as judged by a neuroradiologist: one rrMDD patient had abnormally large ventricles and one rrMDD patient had brain atrophy. Two control participants were excluded because in one a part of the right occipital lobe was missing and in another control a part of the temporal lobe/insula was missing.

## Supplementary Table 1. Mood-ratings

|  | **rrMDD** | | **HC** |  | **Between-group statistics** | | |
| --- | --- | --- | --- | --- | --- | --- | --- |
|  |  |  |  |  |  |  |  |
|  | **Mean** | **SD** | **Mean** | **SD** | **U** | **F** | ***p*** |
| Neutral Mood |  |  |  |  |  |  |  |
| - before scan | 7.30 | 0.86 | 7.58 | 0.71 | 688 |  | 0.23 |
| - after scan | 6.89 | 1.26 | 7.28 | 0.64 | 578 |  | 0.34 |
| - difference^1^ | -0.41 | 0.93 | -0.022 | 0.65 |  | 0.846 | 0.361 |
| Sad Mood |  |  |  |  |  |  |  |
| - before MIP | 6.32 | 1.26 | 6.91 | 0.79 | 461 |  | 0.028 |
| - after MIP | 4.08 | 1.66 | 4.90 | 1.32 | 565 |  | 0.007 |
| - difference^2^ | -2.13 | 1.50 | -2.15 | 1.17 |  | 0.004 | 0.95 |
| - lowest* | 4.99 | 1.88 | 5.94 | 1.39 | 490 |  | 0.027 |

HC: healthy control; MIP: Mood-induction Procedure; rrMDD: remitted recurrent major depressive disorder; U: Mann-Whitney U non-parametric test statistic; F:F-statistic from repeated measures analysis; *p*: *p*-value; SD: Standard Deviation;^1^ Scores significantly decreased during sad resting-state in both groups (p<0.001). ^2^Subjects rated their lowest mood during the sad resting-state scan retrospectively.

## Supplementary Figure 3. Dominant FC-states emerging in brain activity over time

**
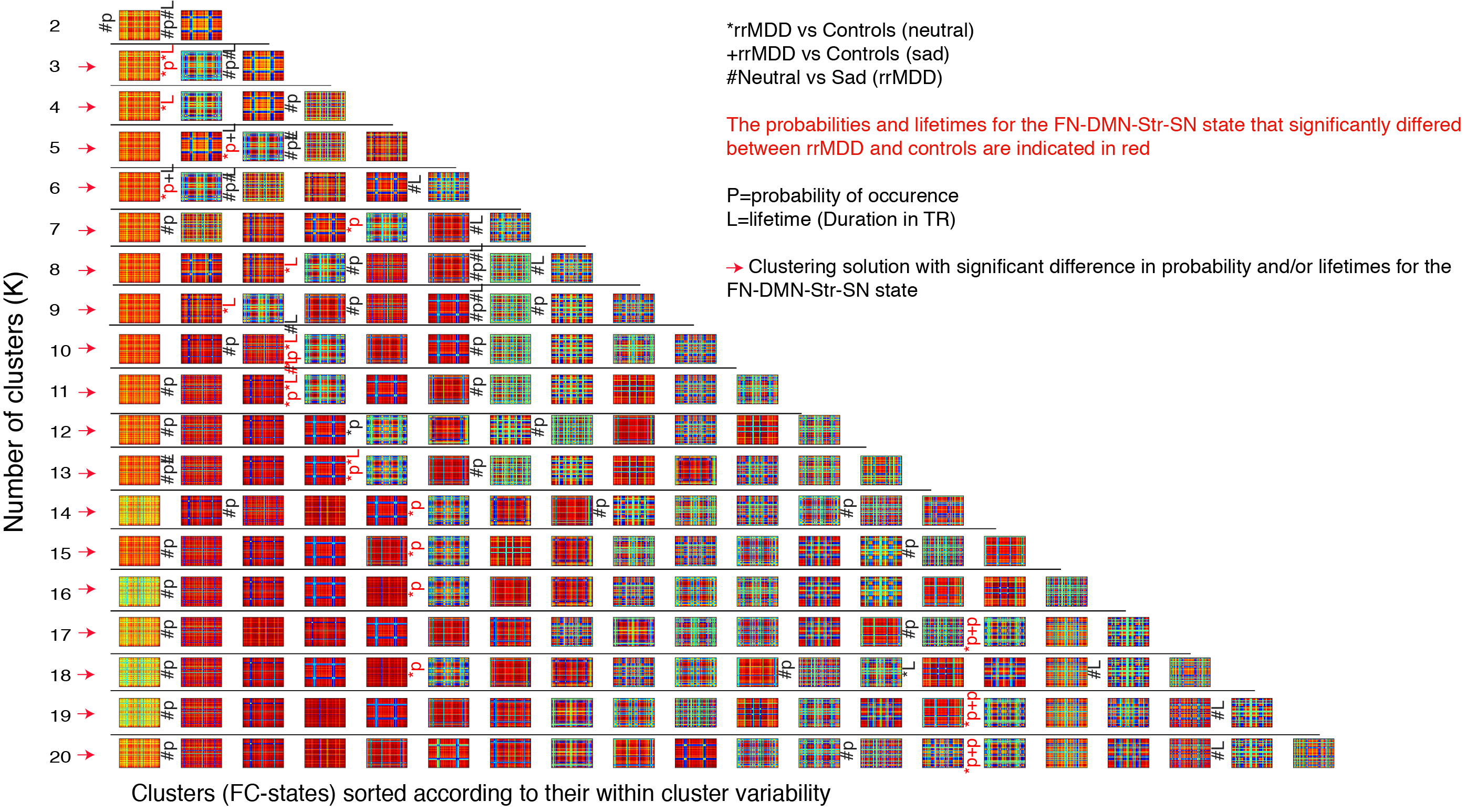
**

**Supplementary Figure 3.** Dominant FC-states emerging in brain activity over time.

We ran the *k*-means clustering algorithm with *k* ranging from 2 to 20 (vertical axis) and for each *k* detected *k* recurrent FC-states, over all time points, over all subjects and both mood-states. Depending on *k*, a more distinct subdivision in different FC-states is identified from the entire sample. A permutation-based 2-sample t-test (with 10000 permutations) was used to identify significant differences between groups (rrMDD patients versus controls) and a permutation based paired T-test (with 10000 permutations) was used to identify differences between mood-states (neutral versus sad mood). Results are corrected for number of states with *p<0.05/k*. FC-states that differ significantly in terms of probability between rrMDD and controls are indicated left of the FC-state with *P in neutral mood (15 states), and in +P, in sad mood (3 states). FC-states that differ in terms of lifetime (duration) are indicated with *L in neutral mood (7 states) +L in sad mood (2 states). In rrMDD, FC-states that differ significantly in terms of probability between neutral vs. sad are indicated with #P for probability (33 states) and #L for mean lifetime (15 states). In controls, no states differed in terms of probability or mean lifetime for neutral vs. sad mood. In red we indicate the FN-DMN-Str-SN state that significantly differs between rrMDD patients and controls in probability of occurrence or duration. Furthermore, with a red arrow we indicate in which clustering solutions the FN-DMN-Str-SN state is significantly different between groups for probability of occurrence and/or duration. As shown in the figure, the FN-DMN-Str-SN state consistently differs between groups, largely independent of which clustering solution is chosen. Abbreviations: rrMDD: remitted-recurrent MDD; DMN: Default Mode Network; SN: Salience Network; Str: striatum; FN: Frontal Network; TR: repetition time

## Supplementary Figure 4. variance in each k-means derived FC-state for each partition model.

**
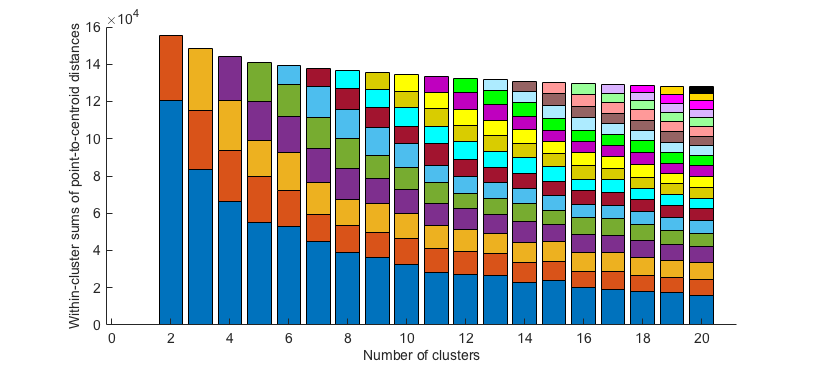
**

**Supplementary Figure 4.** This figure illustrates how much variance there is in each k-means derived state for each partition model. For each partition model from k=2 to 20 (represented by each column) the within cluster sums of point-to-centroid distances (the 4^th^ output of the k-means algorithm in Matlab) is shown. Each cluster (sorted according to their probability of occurrence) is represented by a different colour. As expected, we find that both the within-cluster distance/variability and the total sum of within-cluster distances decrease with increasing the number of clusters. We also find that the first state (the one occurring with more probability, corresponding to the global mode) is always the one with more variability.

## Supplementary Figure 5. Overlap of resting-state networks with the FN-DMN-Str-SN state

**
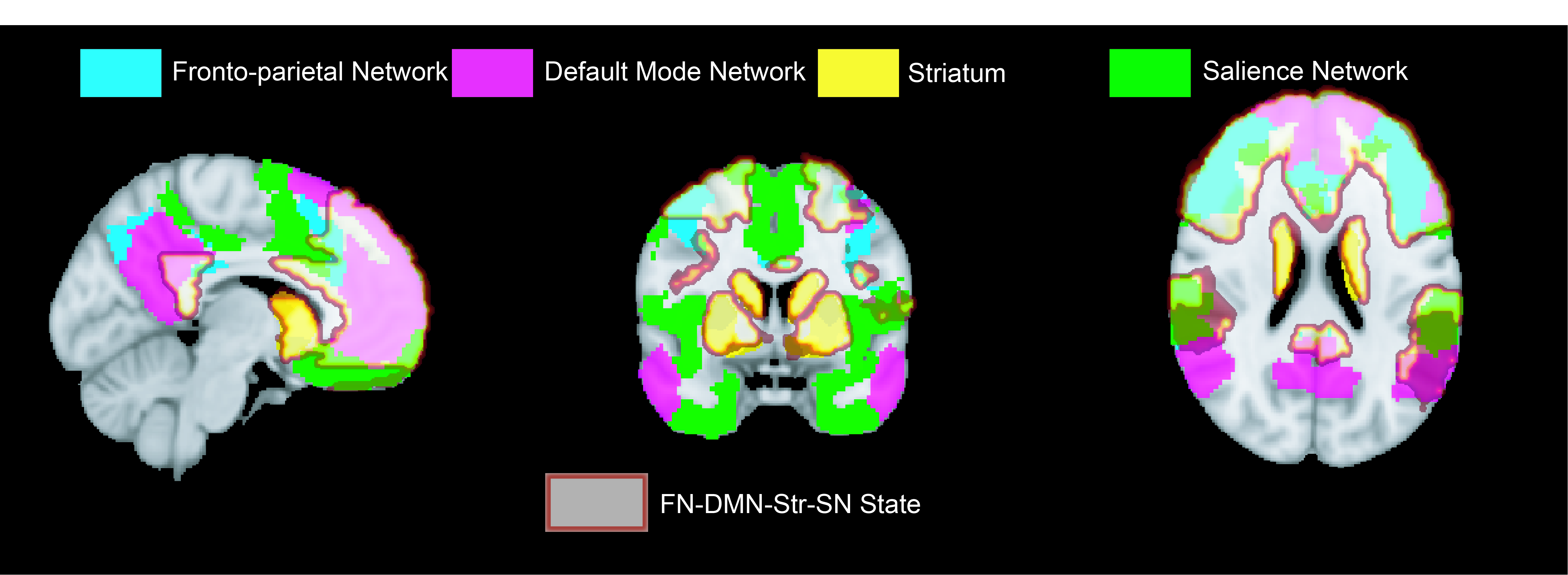
**

**Supplementary Figure 5.** Overlap of resting-state networks with the FN-DMN-Str-SN state. The FN-DMN-Str-SN state is projected on top of 4 resting-state networks, defined according to a cortical 7-network parcellation based on 1000 participants by Yeo et al. (Yeo et al., 2011) and a striatum parcellation by Choi et al. (Choi, Yeo, & Buckner, 2012) to show overlap of this FC-state with traditional resting-state networks. Abbreviations: DMN: Default Mode Network; SN: Salience Network; Str: striatum; FN: Frontal Network

## Supplementary Results

## FC-state analysis for remitted-MDD and controls separately

*Probability of occurrence*

In rrMDD, out of the 19 clustering solutions we considered (with *k* from 2 to 20), in 19 clustering solutions 33 FC-states occurred less (lower probability) in sad mood compared to neutral mood after correcting for the number of states (thus in some clustering solutions, multiple FC-states differed for neutral vs. sad mood in rrMDD). Thirteen of these FC-states consisted of the Insula-Auditory State (**Supplementary Figure 6A**)**,** 15 FC-states consisted of the Somatosensory-Insula state (**Supplementary Figure 6B**)**,** four FC-states consisted of the Visual-Hippocampus state **(Supplementary Figure 6C**). Additionally, one FC-state, the global state, occurred more often **(Supplementary Figure 6D**)**.** For illustrative purposes, we display the states derived from the clustering solution *k*=10 (also see **Figure 3** in the main document for these FC-states for *k*=10).

*Duration*

In rrMDD, out of 19 clustering solutions we considered (with *k* from 2 to 20), 15 FC-states lasted significantly shorter (lower mean lifetime), in sad mood compared to neutral mood after correcting for multiple comparisons. One FC-state consisted of the Insula-Auditory state (**Supplementary Figure 5A**), five FC-states consisted of a Visual-Hippocampus state (**Supplementary Figure 5C**), two FC-states consisted of the FN-DMN-Str-SN state (**Supplementary Figure 5E**)**,** four consisted of the Somatosenory-Insula state and four consisted of a Somatosensory-Visual State (**Supplementary Figure 5F**). For illustrative purposes, we display the states derived from the clustering solution *k*=10 (also see **Figure 3** in the main document for these FC-states for *k*=10).

In controls, there were no differences for probability or mean lifetime for neutral vs. sad mood.

## Supplementary Figure 6. FC-states showing differences in probability/duration in rrMDD


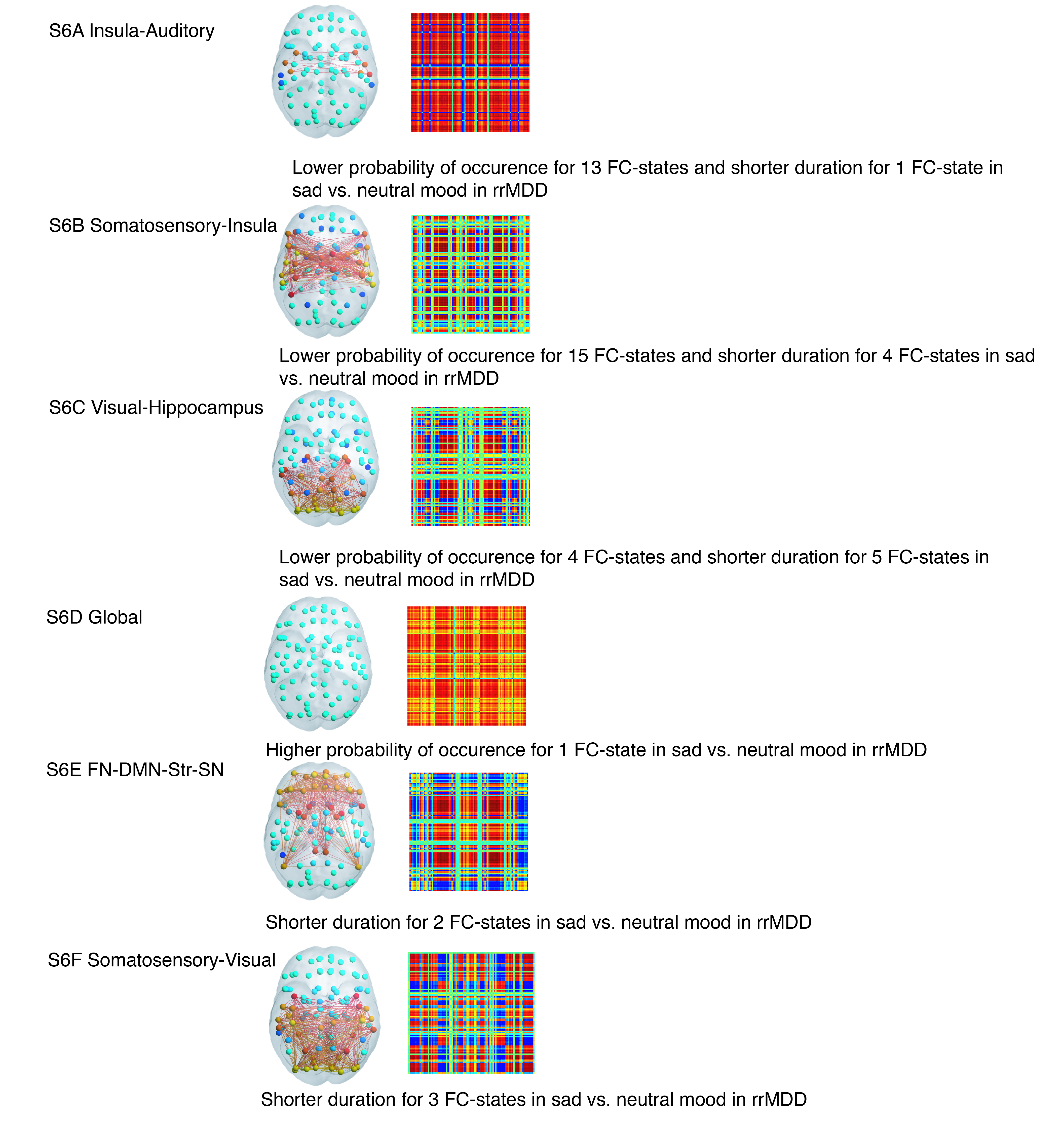


**Supplementary Figure 6.** FC-states showing differences in probability and/or duration for neutral vs. sad mood in rrMDD. For illustrative purposes, FC-states are displayed as derived from the clustering solution *k*=10. Differences were not only observed for *k*=10, but in multiple different clustering solutions (from *k*=2 to 20; **Supplementary Figure 3**). FC-states are represented in the cortical space, where functionally connected brain areas (represented as spheres) are colored alike. The spheres colored in yellow/red represent areas that are all positively correlated between them, but negatively correlated with the rest of the brain (blue colored spheres). FC-states are also represented as the eigenvector’s outer product, which is a 90x90 matrix representing the number of brain areas and red or blue indicate positive or negative BOLD phase synchronization between them. Within group differences were calculated using a permutation based paired T-test with 10000 permutations and we corrected for the number of states (*p*>0.05/k). Abbreviations: rrMDD: remitted-recurrent MDD; DMN: Default Mode Network; SN: Salience Network; Str: striatum; FN: Frontal Network

*Switching profiles for k=10*

For sad compared to neutral mood, rrMDD showed decreased probability to switch from the Somatosensory-Insula state to the Visual-Hippocampus state in the *k*=10 solution, (28% vs. 19%, *p*=0.0028; before correcting for the number of states; Supplementary Figure 8A). Of note, these two FC-states also showed a lower probability of occurrence and duration during sad versus neutral mood in MDD. This might explain the lower switching probabilities between these states in sad compared to neutral mood.

For sad versus neutral mood, controls showed increased probability to switch from the Prefrontal-Limbic state to the global state (8% vs. 4%, *p*=0.0025) and a decreased probability of switching from the Somatosensory-Visual state to the Visual state, (0.6% vs 7.5%, p=0.0046, before correcting for the number of states; **Supplementary Figure 8B)**.

See **Supplementary Figure 7** for all between and within group differences uncorrected for the number of states and **7A/B** for all within group differences in switching probabilities for neutral versus sad mood corrected for multiple comparisons.

## Supplementary Figure 7. Switching probablity differences; matrices


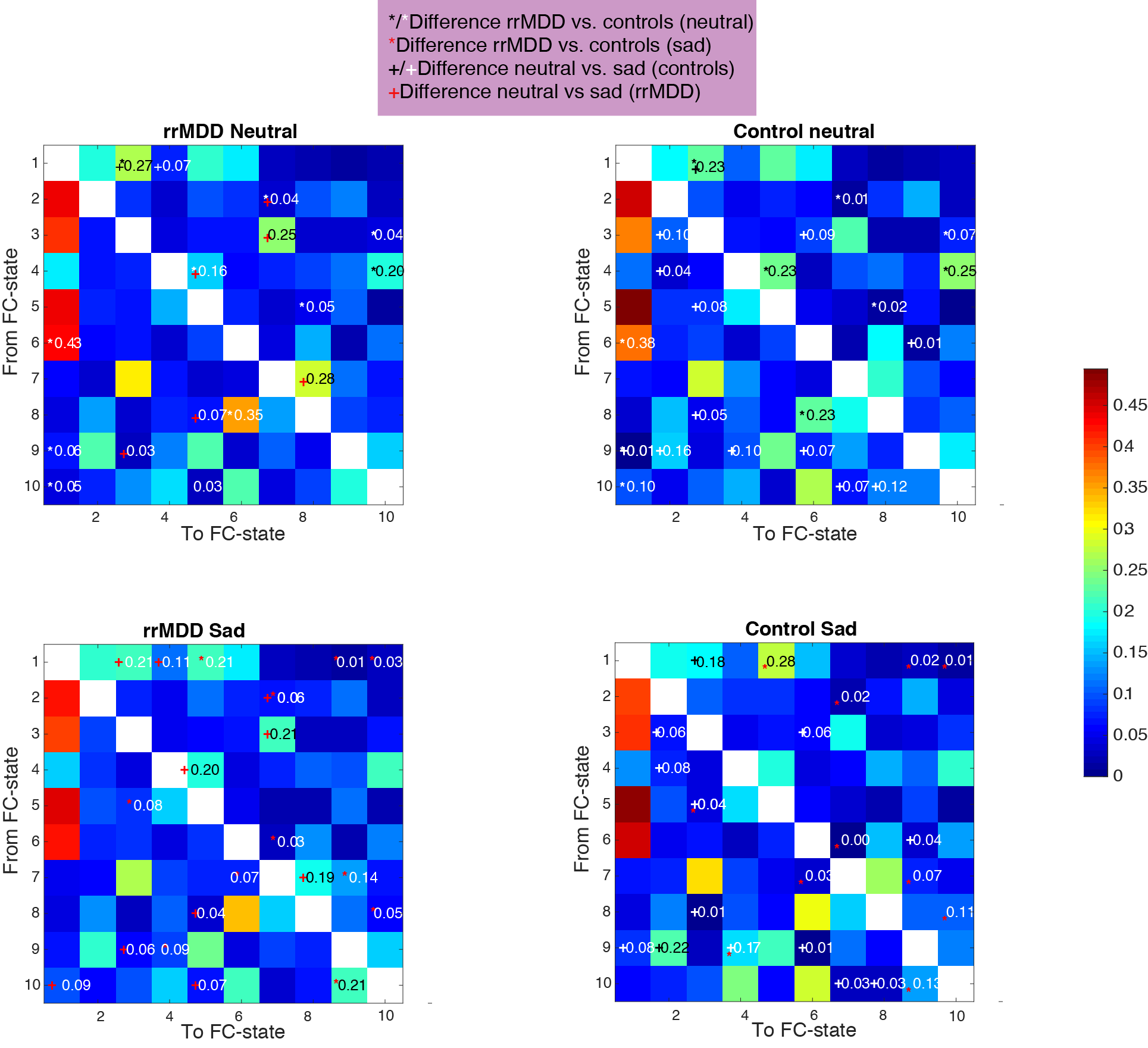


**Supplementary Figure 7.** Switching probablity matrices, showing differences between rrMDD and controls in neutral and sad mood for *k*=10, not corrected for multiple comparisons. Matrices indicate the probability of, given being in a FC state (rows), transitioning to any of the other states (columns). Values indicate number of switches (relative to all switches during the time series) and were estimated for each participant. Transitioning differences between groups were calculated using a permutation based T-tests (paired T-test or 2 sample T-tests with 10000 permutations). All differences are not corrected for multiple comparisons.* differences (*p*<0.05) in probability of transitioning to another state for rrMDD vs controls in neutral mood, * differences (*p*<0.05) in probability of transitioning to another state for rrMDD vs. controls in sad mood + differences in probability of transitioning to another state for neutral vs. sad mood in controls, + differences in probability of transitioning to another state for neutral vs. sad mood in rrMDD. Abbreviations: rrMDD: remitted-recurrent MDD

## Supplementary Figure 8. A/B Switching probability differences; figures

**
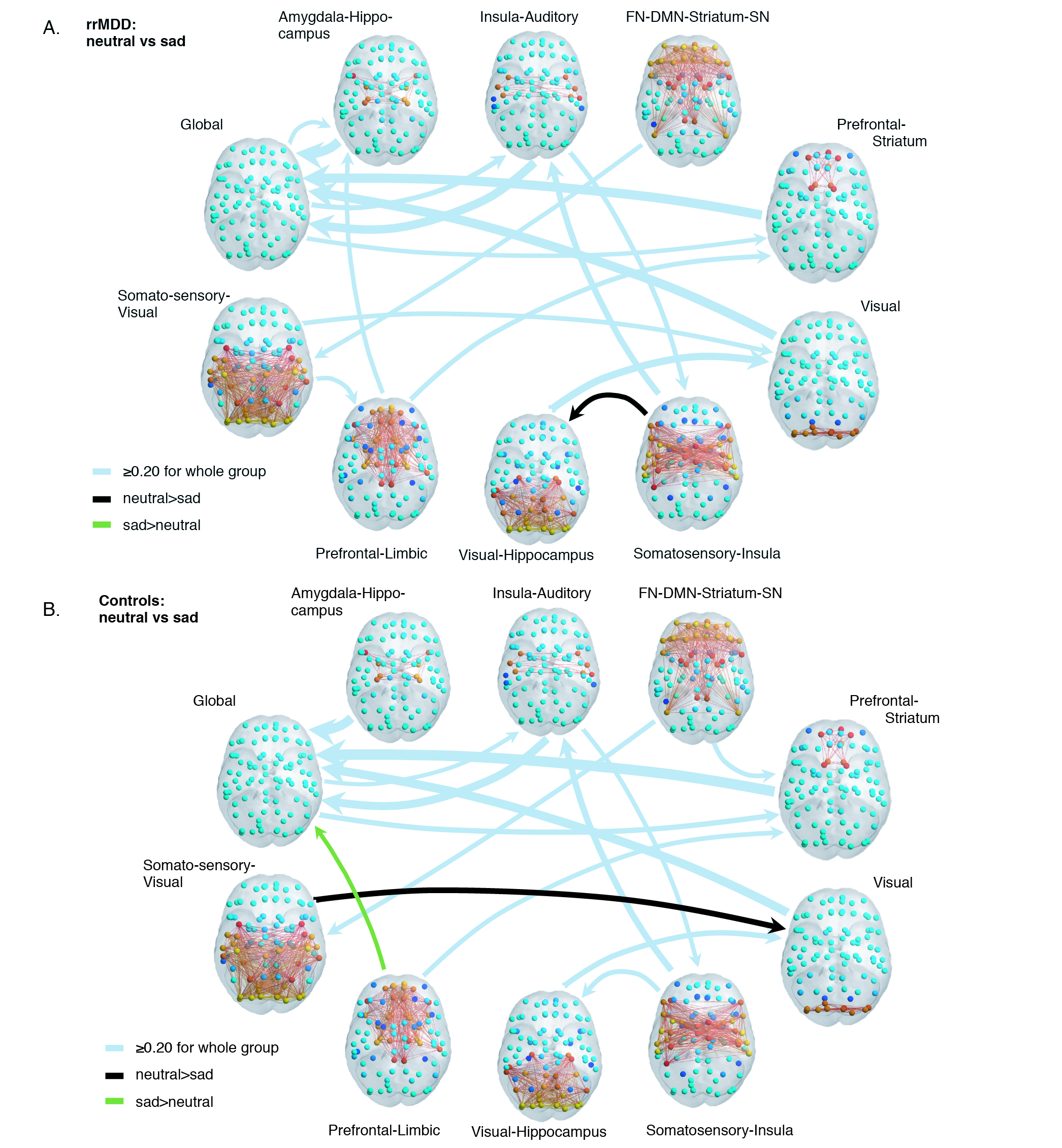
**

**A.**

**B.**

**Supplementary Figure 8.** Differences in probabilities of switching between FC-states for *k*=10, for **A**. rrMDD in neutral vs. sad mood. **B**. Controls in neutral vs. sad mood. Switching probabilities averaged for neutral and sad for groups separately are shown above a threshold of 20% probability of switching to show more frequent switches. The switching matrices (titled ‘rrMDD mean mood’ and ‘control mean mood’) indicate the probability of, being in a given FC-state (rows), transitioning to any of the other states (columns). FC-states are represented in the cortical space, where functionally connected brain areas (represented as spheres) are colored alike. The spheres colored in yellow/red represent areas that are all positively correlated between them, but negatively correlated with the rest of the brain (cyan/blue colored spheres). The light blue arrows from and to the FC-states indicate the switching probabilities averaged over mood state, scaled to the magnitude of probability of switching. Significantly different transitions (*p*<0.05/10) are illustrated in this figure, with black arrows representing the transitions that occur with higher probability in neutral mood and in greens the ones that occur with higher probability in sad mood. Values were estimated for each subject and then a permutation-based paired t-test (10000 permutations) was applied to test for the between-group or within-group significance. Abbreviations: rrMDD: remitted-recurrent MDD; DMN: Default Mode Network; SN: Salience Network; Str: striatum; FN: Frontal Network;

## Supplementary Discussion

## Entropy for k=10 and for the FN-DMN-Str-SN state separately

We tested if there was a significant difference in entropy (Shannon, 1948) between groups for *k*=10, by computing for each subject s, the overall entropy as:

$$H\left( s \right)=-\sum(P_{s}\log_{2} P_{s})$$

where *P_s_* is a vector containing the probabilities of each state for subject s. We tested differences in entropy for *k*=10. Entropy is a measure of the average amount of uncertainty or predictability present in a given probability distribution (Wang, 2008). The higher the homogeneity of probability values across states, the higher the entropy for that subject will be, and the lower the predictability of the occurrence of states. To the extreme, if only one state has 100% probability and the others have null probability, than the entropy is zero. Thus, lower entropy indicates more restricted dynamical repertoire (Carhart-Harris et al., 2014).

Additionally, we investigated the entropy associated with the FN-DMN-Str-SN state, given by

$H_{c}\left( s \right)=-P_{s}(c)\log_{2} P_{s}(c))$

In the case of one state, if a state has high entropy there is high uncertainty/low predictability of occurrence of that state.

We found that, the overall entropy of the system was statistically similar in MDD patients in remission compared to controls for the clustering solution of *k*=10 in neutral mood (mean H(s)=2.59 vs. 2.61 respectively, p=0.63) and in sad mood (mean H(s)=2.48 vs. 2.51 respectively, p=0.26). This indicates that, despite the significant decrease in the probability of the FN-DMN-Str-SN state in remitted patients, the distribution of probabilities is not affected, which suggests a regulation by intrinsic homeostatic processes. Yet, we found that the entropy associated with the FN-DMN-Str-SN state was significantly decreased in rrMDD patients compared to controls (mean H(c)=0.19 vs. 0.25 respectively, p=0.006) in neutral mood. In sad mood however, we found no significant differences between rrMDD and controls in entropy of this FC-state (mean H(c)=0.23 vs. 0.26 respectively, *p*=0.16).

This finding indicates high predictability/less uncertainty of probability of occurrence values of this FC state in remitted MDD patients in neutral mood. This might be associated with the flexibility with which this state occurs and thus supports the idea that the flexibility of brain dynamics is reduced in psychiatric disorders, including MDD (Jin et al., 2017).

## References

Carhart-Harris, R., Leech, R., Hellyer, P., Shanahan, M., Feilding, A., Tagliazucchi, E., . . . Nutt, D. (2014). The entropic brain: a theory of conscious states informed by neuroimaging research with psychedelic drugs. *Front Hum Neurosci, 8*(20). doi:10.3389/fnhum.2014.00020

Choi, E. Y., Yeo, B. T., & Buckner, R. L. (2012). The organization of the human striatum estimated by intrinsic functional connectivity. *J Neurophysiol, 108*(8), 2242-2263. doi:10.1152/jn.00270.2012

Jin, C., Jia, H., Lanka, P., Rangaprakash, D., Li, L., Liu, T., . . . Deshpande, G. (2017). Dynamic brain connectivity is a better predictor of PTSD than static connectivity. *Hum Brain Mapp, 38*(9), 4479-4496. doi:10.1002/hbm.23676

Mocking, R. J. T., Figueroa, C. A., Rive, M. M., Geugies, H., Servaas, M. N., Assies, J., . . . Ruhé, H. G. (2016). Vulnerability for new episodes in recurrent major depressive disorder: protocol for the longitudinal DELTA-neuroimaging cohort study. *BMJ Open, 6*(3), e009510. doi:10.1136/bmjopen-2015-009510

Shannon, C. E. (1948). A mathematical theory of communication (parts I and II). *Bell System Tech. J., 27*, 379-423.

Wang, Q. A. (2008). Probability distribution and entropy as a measure of uncertainty. *Journal of Physics A: Mathematical and Theoretical, 41*(6), 065004.

Yeo, B. T., Krienen, F. M., Sepulcre, J., Sabuncu, M. R., Lashkari, D., Hollinshead, M., . . . Buckner, R. L. (2011). The organization of the human cerebral cortex estimated by intrinsic functional connectivity. *J Neurophysiol, 106*(3), 1125-1165. doi:10.1152/jn.00338.2011

(Mocking et al., 2016)
